# Mapping the global interactome of the ARF family reveals spatial organization in cellular signaling pathways

**DOI:** 10.1242/jcs.262140

**Published:** 2024-05-14

**Authors:** Laura Quirion, Amélie Robert, Jonathan Boulais, Shiying Huang, Gabriela Bernal Astrain, Regina Strakhova, Chang Hwa Jo, Yacine Kherdjemil, Denis Faubert, Marie-Pier Thibault, Marie Kmita, Jeremy M. Baskin, Anne-Claude Gingras, Matthew J. Smith, Jean-François Côté

**Affiliations:** ^1^ Montreal Clinical Research Institute (IRCM), Montréal, QC H2W 1R7, Canada; ^2^ Molecular Biology Programs, Université de Montréal, Montréal, QC H3T 1J4, Canada; ^3^ Department of Chemistry and Chemical Biology and Weill Institute for Cell and Molecular Biology, Cornell University, Ithaca, NY 14853, USA; ^4^ Institute for Research in Immunology and Cancer, Université de Montréal, Montréal, QC H3T 1J4, Canada; ^5^ Department of Medicine, Université de Montréal, Montréal, QC H3C 3J7, Canada; ^6^ Department of Experimental Medicine, McGill University, Montréal, QC H3G 2M1, Canada; ^7^ Lunenfeld-Tanenbaum Research Institute, Sinai Health System, Toronto, ON M5G 1X5, Canada; ^8^ Department of Molecular Genetics, University of Toronto, Toronto, ON M5S 1A8, Canada; ^9^ Department of Anatomy and Cell Biology, McGill University, Montréal, QC H3A 0C7, Canada

**Keywords:** ARF GTPases, BioID proteomics, ARF-like GTPases, ARLs, Effector proteins, ESCPE-1, PLD1

## Abstract

The ADP-ribosylation factors (ARFs) and ARF-like (ARL) GTPases serve as essential molecular switches governing a wide array of cellular processes. In this study, we used proximity-dependent biotin identification (BioID) to comprehensively map the interactome of 28 out of 29 ARF and ARL proteins in two cellular models. Through this approach, we identified ∼3000 high-confidence proximal interactors, enabling us to assign subcellular localizations to the family members. Notably, we uncovered previously undefined localizations for ARL4D and ARL10. Clustering analyses further exposed the distinctiveness of the interactors identified with these two GTPases. We also reveal that the expression of the understudied member ARL14 is confined to the stomach and intestines. We identified phospholipase D1 (PLD1) and the ESCPE-1 complex, more precisely, SNX1, as proximity interactors. Functional assays demonstrated that ARL14 can activate PLD1 *in cellulo* and is involved in cargo trafficking via the ESCPE-1 complex. Overall, the BioID data generated in this study provide a valuable resource for dissecting the complexities of ARF and ARL spatial organization and signaling.

## Teaser

**Summary:** Generation of the ARF family interactome allowed the attribution of potential localizations and functions to previously understudied members. ARL14 was found to activate PLD1 and contribute to ESCPE-1-mediated trafficking.

## INTRODUCTION

The ADP-ribosylation factor (ARF) family of small GTPases belongs to the RAS superfamily, together with RHO, RAS, RAN and RAB proteins. ARFs participate in a plethora of cellular processes including membrane trafficking, modulation of membrane lipid composition and cytoskeleton remodeling (Sztul et al., 2019). Operating as GTPases, they are presumed to cycle between an inactive GDP-bound state and an active GTP-bound state. This cycling is orchestrated by guanine nucleotide exchange factors (GEFs), which promote GDP dissociation to allow GTP binding, and GTPase-activating proteins (GAPs), which enhance the intrinsic GTP hydrolysis activity (Sztul et al., 2019; Bourne et al., 1991). Unlike other RAS proteins that undergo lipidation at the C-terminus, most ARFs have a distinctive N-terminal extension modified with various lipid groups that forms an amphipathic helix (Gillingham and Munro, 2007). This helix is buried in a hydrophobic pocket in inactive ARFs and is inserted into membranes concurrent with GTP loading (Amor et al., 1994; Antonny et al., 1997). This conformational change places ARFs closer to membranes than other RAS proteins, creating a unique signaling environment (Gillingham and Munro, 2007). As ARF coupling to effectors occurs on membranes of complex lipid compositions and of various curvatures, capturing these events biochemically is a limiting step into our current understanding of ARF signaling (Sztul et al., 2019).

The ARF family consists of 29 members, categorized into five classical ARFs (class I: ARF1 and ARF3; class II: ARF4 and ARF5; and class III: ARF6), two secretion-associated RAS proteins (SAR1A and SAR1B) (Nachury et al., 2007), 21 ARF-like proteins (ARLs) and the tripartite motif-containing protein 23 (TRIM23) (Gillingham and Munro, 2007). Classical ARFs recruit coat proteins that initiate vesicle formation, facilitating membrane trafficking and cargo transport (Tan and Gleeson, 2019). For instance, ARF1 recruits the coat protein complex I (COPI) at the Golgi membrane, enabling vesicular transport to the endoplasmic reticulum (ER) (Palmer et al., 1993). Additionally, ARF1 coordinates the formation of vesicles at the Golgi by recruiting the adaptor proteins AP-1, AP-3 or AP-4 (Dittie et al., 1996; Ooi et al., 1998; Hirst et al., 1999) and GGA proteins (Puertollano et al., 2001) for vesicular trafficking to endosomes or the plasma membrane. Although individual knockdowns of ARF3, ARF4 or ARF5 lack major detectable phenotypes in cultured cells, their co-depletion with ARF1 revealed specific Golgi functions, underscoring a high degree of interplay in ARF-regulated pathways (Volpicelli-Daley et al., 2005). In contrast, ARF6 predominantly operates at the cell periphery where it regulates endocytosis, endosomal recycling and cytoskeletal dynamics, mainly through lipid modification by activating phosphatidylinositol 4-phosphate 5-kinase (Honda et al., 1999), and phospholipase D1 (PLD1) (Toda et al., 1999), or through actin remodeling via activation of RHOA and RAC1 (Santy et al., 2005; D'Souza-Schorey and Chavrier, 2006). Similarly to ARF1, SAR1A and SAR1B play crucial roles in ER-to-Golgi transport by recruiting the coat protein complex II (COPII) and facilitating vesicle budding (Saito et al., 2017; Jones et al., 2003). As paralogues, SAR proteins exhibit both overlapping and unique functions, attributed to differences in enzymatic and effector-binding activities (Melville et al., 2020).

The understudied ARLs control a wide array of biological functions across diverse cellular compartments (Kahn et al., 2006). Notably, ARL8A and ARL8B localize to lysosomes, facilitating their motility (Hofmann and Munro, 2006). Several ARLs, including ARL2, ARL3, ARL4D, ARL6 and ARL13B, play regulatory function in microtubule-associated processes within centrosomes, spindles, midbodies, basal bodies and cilia (Yong et al., 2020; Zhou et al., 2006; Bhattarai et al., 2019; Lin et al., 2020; Nachury et al., 2007; Li et al., 2010). The heterogeneous spatial distribution of some ARLs can drive interplay with multiple cellular processes. For example, ARL2 interacts with β-tubulin and the tubulin chaperone TBCD, promoting cytosolic biogenesis of α/β-tubulin dimers (Francis et al., 2017a,b). Additionally, ARL2 localizes to the mitochondrial intermembrane space, where it drives mitochondrial fusion (Newman et al., 2017). The intricate biological functions of individual ARLs are complicated by their interplay with other ARFs. ARL1 crosstalks with ARF1 and ARF3, influencing the recruitment of the GEF BIG1 at sorting endosomes (D'Souza et al., 2014). Likewise, ARL13B acts as a GEF for ARL3, orchestrating the delivery of cargos into the cilium in cooperation with the ARF GAP RP2 (Gotthardt et al., 2015; Veltel et al., 2008). The effectors of ARLs are still largely unknown, highlighting the necessity of elucidating their cellular functions (Sztul et al., 2019). Moreover, there are still few data describing the biochemical properties underlying ARL nucleotide cycling or their propensity to function as archetypal ‘switch-like’ GTPases. Many ARLs, including ARL5C, ARL9, ARL10, ARL11, ARL13A, ARL14 and ARL16, have yet to be assigned distinct biological functions. Clearly, new approaches are needed to access unexplored layers of ARF and ARL spatial signaling, as well as to uncover their associated proteins in cells.

Defective ARF/ARL signaling contributes to numerous human diseases. Many genetic diseases, known as ‘coatopathies’, are linked to mutations in coat complexes, including in COPI (Watkin et al., 2015; DiStasio et al., 2017; Izumi et al., 2016), adaptor protein complexes (Montpetit et al., 2008; Nesbit et al., 2013; Assoum et al., 2016; Abou Jamra et al., 2011; Slabicki et al., 2010), COPII (Boyadjiev et al., 2006; Bianchi et al., 2009; Garbes et al., 2015) and retromer complex-associated proteins (Gustavsson et al., 2015; Damseh et al., 2015). Additionally, mutations in ARF GEFs have been identified (Sheen et al., 2004). In cancer, disruption of ARF-controlled membrane trafficking promotes cell invasion and migration (Casalou et al., 2020), whereas defective effector signaling can enhance oncogenic signals, such as ARF5- or ARF6-driven activation of PI3K and AKT (Nacke et al., 2021). Finally, pathogens also exploit ARFs or their effectors (Jimenez et al., 2016; Zhou et al., 2022; Mirabelli et al., 2023).

To gain fundamental knowledge into the ARF/ARL signaling pathways and their relevance to cell biology, we performed proximity-dependent biotin identification (BioID) coupled with mass spectrometry (MS) on 28 constitutively active human ARF family members to define the comprehensive ARFome. Our approach reveals the broad landscape of ARF/ARL proximal interactors and defines specificity for GAPs. Leveraging the ability of BioID to provide a history of protein interactions, we were able to interrogate the localization of candidate proteins to define ARF/ARL functional localization. Finally, we discovered a potential coat complex recruited via the newly identified PLD1 activator ARL14 on endosomes, facilitating retrograde traffic.

## RESULTS

### Development of a BioID pipeline to define the ARF family proximity interaction network

**Fig. 1.**
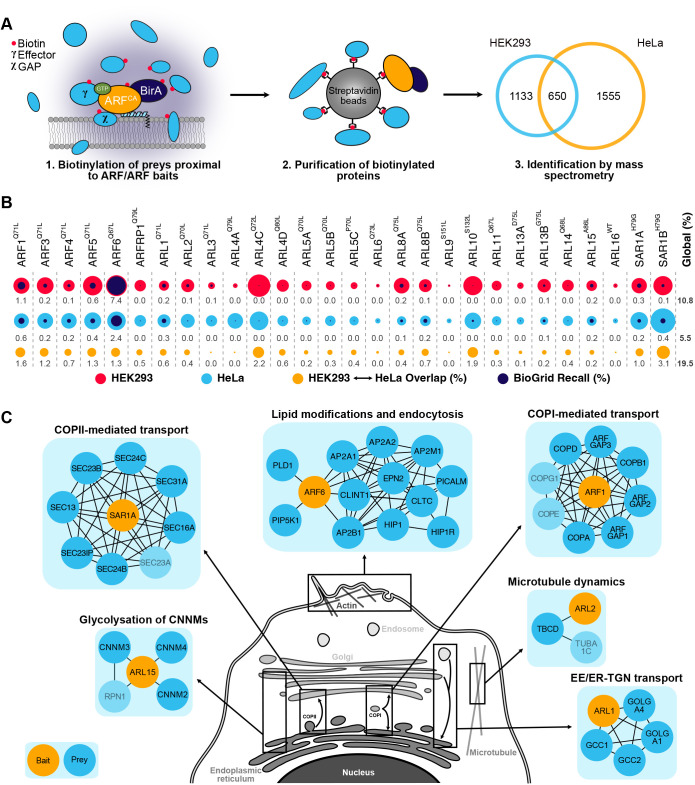
**The ARF proximity interaction network defined by BioID.** (A) Workflow of the proximity-dependent biotin identification (BioID) performed on constitutively active (CA) ARF family baits fused to BirA*–Flag. The Venn diagram illustrates unique and shared interactions between cell lines. (B) Proportional area chart illustrates interactions for each bait in HEK293 (red) and HeLa (light blue) cells, overlapped with those reported in the literature (dark blue) and proportionate to the total interactions in both cell lines. Overlap between cell lines (orange) is also represented. Numbers denotes the overlap percentage. The total overlap and recall percentages are presented under the global column. (C) Known interactors of ARF/ARL GTPases identified by BioID. Interactors depicted in lighter blue were detected but did not pass our stringent thresholding. EE, early endosomes; ER, endoplasmic reticulum; TGN, *trans*-Golgi network.

ARFs and ARLs are dynamic proteins that rely on a unique N-terminal region for membrane proximity, crucial for ARF/ARL signaling. Capturing many ARF-mediated interactions using classical biochemical approaches in the absence of a phospholipid scaffold would be challenging. Instead, we elected to use BioID, a powerful method that has proven effective in systematically studying RHO GTPases in their native environment (Bagci et al., 2020). BioID utilizes an abortive biotin ligase derived from *Escherichia coli* that, when fused to a protein of interest, labels proximal interactors with biotin within a 10 nm radius (Roux et al., 2012). Proteins irreversibly tagged with biotin are affinity purified with streptavidin and identified by MS at high throughput (Nahle et al., 2022). We systematically performed BioID following 24 h of labeling with 28 out of 29 ARF family members as baits. TRIM23 was not included in the study. We chose to insert the BirA* tag at the C-terminal end of ARF/ARL proteins to leave the N-terminal extension unmodified (Fig. 1A).

To enrich for effectors and GAPs, as was previously demonstrated for RHO proteins (Bagci et al., 2020), we generated constitutively active mutants of ARFs and ARLS by mutating the glutamine to leucine in the highly conserved G-3 motif (WDXGGQ), which prevents GTP hydrolysis. There are no *in vitro* biochemical data describing nucleotide cycling for most ARFs, but early work on ARF1 determined that a Q71L mutation disrupted GTP hydrolysis in a manner similar to that of RAS (Kahn et al., 1995). However, in contrast to the classical RAS GTPases, the mutation in ARF1 did not alter the rate of nucleotide release or relative affinity to GDP or GTP. This suggests thar the ‘activating’ mutation drives only a low-level increase in GTP loading, yet this remains valuable for interactome mapping. A 24-h labeling time is thus advantageous to identify a comprehensive set of binding partners. For GTPases lacking the conserved glutamine (ARL5C, ARL9, ARL10, ARL13A, ARL15, SAR1A and SAR1B), we generated specific mutants based on the position where the glutamine would normally be found. ARL16 was kept in its wild-type (WT) form as the G-3 motif is atypical (RELGGC) (Dewees et al., 2022) and its biochemical properties are unknown.

Because ARF nucleotide cycling and the impact of RAS^Q61L^-derived mutations are largely unstudied, we performed and compared BioID interactomes for ARF1, ARF4, ARF6 and SAR1A in both their WT and active states (Table S7). This revealed a significant increase in the abundance of known effectors and GAPs when using the active mutants. Conversely, GEFs were enriched with WT baits, consistent with the knowledge that they preferentially interact with the GDP-bound GTPases (Fig. S1). These results support the use of active ARF/ARL mutants for identification of binding partners, though their behavior might differ from that of RAS and RHO GTPases.

To establish the experimental controls, we used BirA*–Flag and BirA*–Flag–eGFP to selectively filter out proteins biotinylated at random. Likewise, BirA*–Flag–eGFP–CAAX served as a control targeted to membranes, which filtered for membranous preys with less than a 1.7-fold enrichment of a bait in comparison to this control. All baits used in this study are listed in Table S1. To ensure broad coverage and confidence in the proximity interactomes, we conducted BioID in two cell lines. We used Flp-In T-Rex HEK293 as a classical model for proteomics and Flp-In T-Rex HeLa for its oncogenic profile and for convenience of imaging. Using inducible cell lines mitigates potential long-term cellular damage caused by the expression of the ARFs/ARLs.

We conducted validation of both the inducible expression and the biotinylation capacity of all baits by western blotting (Fig. S2). Additionally, we assessed the subcellular localization of each bait by immunofluorescence (Fig. S3). Co-staining experiments involving well-characterized ARF family members were performed to ensure that the tagging did not affect their localizations. Our results confirmed the localization of ARF1, ARF3, ARF4 and ARF5 at the Golgi (Fig. S4A,B), whereas ARL8A and ARL8B exhibited colocalization with lysosomes (Fig. S4C). Also, the ER membrane-resident SAR1A and SAR1B displayed a network-like immunostaining pattern adjacent to KDEL immunostaining, marking these proteins as targeted to the ER lumen (Fig. S4D).

### Using BioID to systematically delineate the ARFome

We systematically conducted BioID screens for all active baits, identifying over 63,000 proximity interactions (Table S8). Following stringent filtering, we uncovered 1783 high-confidence interactors in HEK293 cells and 2205 in HeLa cells, with 650 proximity interactors shared between the two cell lines (Fig. 1A). The non-shared interactions between the cell lines might be due to the different protein expression profiles, stringent thresholding and variations in the sensitivity of the two mass spectrometers used for HEK293 and HeLa cells. The overlapping preys for each bait between the two cell lines along with a comparison to known interactions from the literature are depicted in Fig. 1B. Overall, approximately 10% of our hits in HEK293 cells and 6% in HeLa cells overlap with those from existing literature (Fig. 1B).

The quality of our BioID screens was benchmarked by leveraging previously established interactions. Within these interactions, the ARF1 dataset featured GAPs and the COPI complex (Fig. 1C). Additionally, the complete COPII complex was identified within the SAR1A dataset (Fig. 1C). ARF6 BioID retrieved known interactors involved in lipid composition modulation and endocytosis (Fig. 1C). Furthermore, ARL1 identified the four metazoan GRIP domain golgins, which require ARL1 for their targeting to the Golgi (Setty et al., 2003; Panic et al., 2003) (Fig. 1C). ARL2 was found proximal to TBCD and β-tubulin, known to form a trimer important for maintaining microtubule densities (Francis et al., 2017b) (Fig. 1C). ARL15 acts as a negative regulator of cellular Mg^2+^ transport by modulating N-glycosylation of CNNM proteins, with our BioID hits including CNNM2, CNNM3, CNNM4 and ribophorin I protein (RPN1) (Zolotarov et al., 2021) (Fig. 1C). The recall of these interactions underscores the quality of the BioID screens, providing confidence in the potential to reveal new protein interactions and uncovering unsuspected functions for both extensively studied and less explored ARF/ARL proteins. The comprehensive BioID data are available for exploration by the research community at http://prohits-web.lunenfeld.ca.

### Mapping the functional localization of ARF and ARL proteins *in cellulo* using BioID

**Fig. 2.**
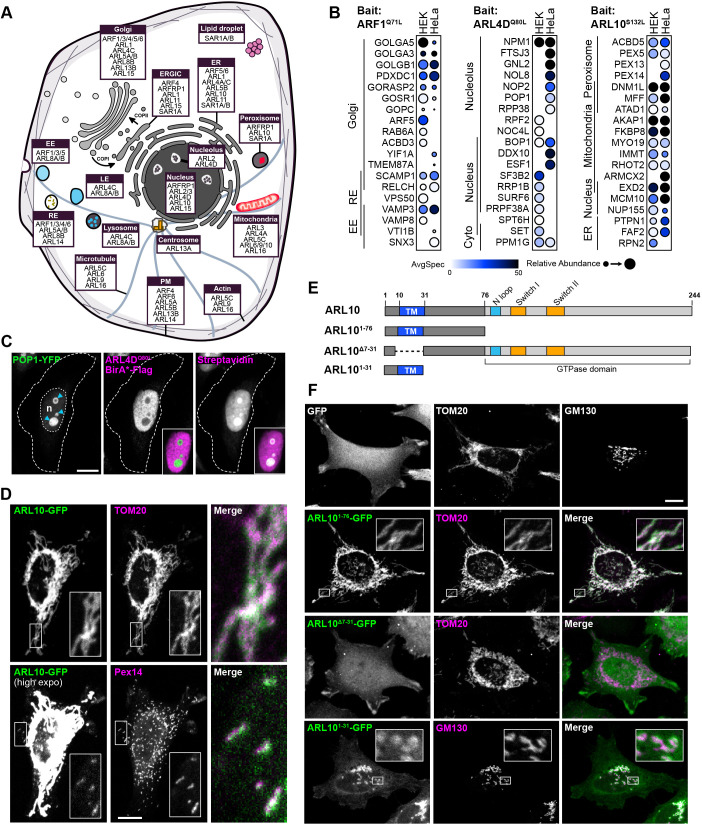
**ARF location assignment from BioID results.** (A) Illustration of the top three cellular compartments (top four in case of ties) enriched for each ARF/ARL as assigned by Human Cell Map. (B) Dotplots presenting the preys enriched in the top three cellular compartments identified with Human Cell Map for the indicated active baits. Darker circles represent higher spectral counts and circle size shows relative abundance. Average probability ≥0.95. AvgSpec, average spectral count. (C) Flp-In HeLa cells expressing POP1–YFP and active ARL4D–BirA*–Flag were stained with anti-Flag and Alexa Fluor 633-conjugated streptavidin (blue arrowheads, nucleoli; n, nucleus). Scale bar: 10 µm. (D) Co-immunostaining of Flp-In HeLa cells expressing ARL10^WT^–GFP with anti-TOM20 (mitochondria marker) and anti-PEX14 (peroxisome marker). Scale bar: 10 µm. (E) Schematic representations of the ARL10 fragments used in this study. The N-terminal region (dark gray) includes the putative transmembrane (TM) domain (blue) and the ARF GTPase domain (light gray) includes the N-loop, switch I and switch II domains. (F) Immunostaining with anti-TOM20 or anti-GM130 (Golgi marker) of Flp-In HeLa cells expressing the indicated chimeric proteins. Scale bar: 10 µm. Images are representative of *n*=3 independent experiments. EE, early endosomes; ER, endoplasmic reticulum; ERGIC, ER–Golgi intermediate compartment; LE, late endosomes; PM, plasma membrane; RE, recycling endosomes.

Accurate determination of the localizations and the intricate vesicular trafficking between intracellular compartments regulated by ARFs and ARLs has proven challenging using biochemical approaches or epifluorescence microscopy. Using BioID with a 24-h labeling window enables the capture of a global overview of the proximal protein interactions occurring at specific subcellular locations during dynamic cellular processes. Hence, mapping the spatial distribution of ARF/ARL interaction with their effectors and their regulators holds great potential in deciphering additional molecular functions of ARFs/ARLs within specific organelles. Exploiting our BioID data, our aim was to assign to each bait its main intracellular localizations by querying the Human Cell Map database, a BioID-based online tool that compared 192 subcellular markers to predict bait localization (Go et al., 2021). Using this resource, we systematically predicted the top three functional localizations of each ARF and ARL (Fig. 2A). From our BioID results, at least one previously reported localization for most well-studied baits was identified. As proof of concept, analyses of ARF1 BioID indicated a localization to the Golgi, corroborated by the presence of various golgins (GOLGA3, GOLGA5 and GOLGB1). Additionally, the tool suggested localization of ARF1 to recycling endosomes, where VPS50, part of the EARP complex, was identified (Stearns et al., 1990; Kondo et al., 2012). The third localization predicted by the ARF1 BioID analyses was the early endosome from the presence of SNARE proteins (VAMP3, VAMP8 and VTI1B) (Fig. 2A,B). However, BioID data were unable to predict the expected localization for ARFRP1, ARL4A and ARL11. The recapitulation of previously reported localizations for most well-studied bait validated the efficacy of this approach. Therefore, mapping the localizations of ARF/ARL proteins could unveil new layers of information.

Leveraging the information provided by the ARF family localization mapping described above, we examined the functional distributions of the atypical ARL4D, given that multiple subcellular compartments are reported in the literature. ARL4D undergoes myristoylation at its N-terminus,which is crucial for membrane anchorage (Li et al., 2007), whereas a short basic extension at its C-terminus serves as a nuclear localization signal (Jacobs et al., 1999) (Fig. S5A). Consequently, previous studies documented the localization of ARL4D at the plasma membrane, cytoplasm and nucleus (Li et al., 2007). Using the BioID data on ARL4D, the Human Cell Map analyses indicated predominant localizations to the nucleus, cytoplasm and nucleolus (Fig. 2A,B). Co-immunostaining of ARL4D with POP1, a nucleolus-resident protein exhibiting proximity to ARL4D according to the BioID (Fig. 2B), revealed exclusion of ARL4D from the nucleolus. However, biotinylated preys displayed pronounced colocalization with POP1 in the nucleolus (Fig. 2C). This result hints at a potential role for ARL4D at the interface between the nucleoplasm and the nucleolus, which would potentially be overlooked based strictly on ARL4D immunostaining.

Determining the nucleotide-loaded state of small GTPases in cells is extremely challenging and currently undetermined for all ARF family members, including ARL4D. To address this, we adopted an ion-pair reversed-phase high-performance liquid chromatography (IP-RP-HPLC) approach developed to resolve GDP/GTP loading of RAS proteins (Araki et al., 2021). The well-studied KRAS served as a control, and substantial GTP loading of the KRAS^Q61L^ mutant was detected following its purification from HEK293T cells (59% GTP; Fig. S6A,B). WT KRAS remained GDP loaded, as did WT ARF1 (Fig. S6C), consistent with early HPLC results obtained using bacterially purified ARF1 (Randazzo and Kahn, 1994). Supporting the premise that an ARF1^Q71L^ mutant does not spontaneously load GTP (Kahn et al., 1995), this variant did not co-purify with any detectable nucleotide, implying that the mutation might fundamentally weaken nucleotide affinity. To resolve the nucleotide loading in ARL4D, we purified both the WT protein and the Q80L mutant with C-terminal eGFP tags from HEK293T cells. Both variants were purified with no bound nucleotides (Fig. S6D). This is consistent with *in vitro* data showing ARL4A and ARL4C rapidly release nucleotides and are likely purified in the apo state (Jacobs et al., 1999), and explains why the Q80L mutant of ARL4D does not augment binding to some partners (Li et al., 2007). More data are required to fully understand nucleotide loading of ARFs/ARLs and the impact of RAS-mimetic mutations, although ARFs/ARLs have proven difficult to study *in vitro*.

For understudied ARLs, localization had not been previously assigned to ARL5C, ARL9 and ARL10. The BioID data suggest that ARL5C and ARL9 function within mitochondria, as well as in both actin and microtubule cytoskeletons (Fig. 2A). The Human Cell Map predicts that ARL10 accumulates within mitochondria and peroxisomes, as well as in the ER and nucleus (Fig. 2A,B). Co-immunostaining of ARL10–GFP with the mitochondrial import receptor subunit TOM20 (also known as TOMM20) confirmed a mitochondrial localization (Fig. 2D). Moreover, high exposure of ARL10–GFP signals validated the presence of ARL10 in PEX14-positive peroxisomes (Fig. 2D), thus unveiling two new localizations for this unstudied GTPase. Instead of the classical N-terminal extension, ARL10 features a 76 amino acid sequence preceding the GTPase domain, with no homology to other ARF/ARL proteins (Fig. S5A). To investigate whether this region encompasses a mitochondria-targeting signal, we generated a chimera by fusing amino acids 1–76 of ARL10 to GFP. The addition of ARL10^1–76^ proved sufficient to tether GFP to the mitochondria, as evidenced by its colocalization with TOM20 (Fig. 2E,F). Analysis of the ARL10 N-terminal sequence using the membrane insertion prediction tool TMHMM (Krogh et al., 2001) suggested the presence of a putative transmembrane domain spanning amino acids 10–31. This region is primarily composed of hydrophobic residues and is followed by a stretch of polybasic residues, a feature often observed in the N-termini of signal-anchored mitochondrial outer membrane proteins (Fig. S5A) (Walther and Rapaport, 2009). Deletion of the predicted transmembrane domain resulted in the loss of ARL10 mitochondrial localization (Fig. 2E,F). However, a chimera of the first 31 amino acids of ARL10 fused to GFP was not sufficient to induce mitochondrial translocation (Fig. 2F). Instead, ARL10^1–31^–GFP accumulated in the Golgi, as evidenced by its partial colocalization with the Golgi marker GM130 (also known as GOLGA2) (Fig. 2F). Taken together, these findings suggest that ARL10 is unique within the ARF family by the presence of a transmembrane domain, which cooperates with an adjacent polybasic residue for its targeting to the outer membrane of mitochondria.

### Mapping the ARF and ARL effectors and regulators

**Fig. 3.**
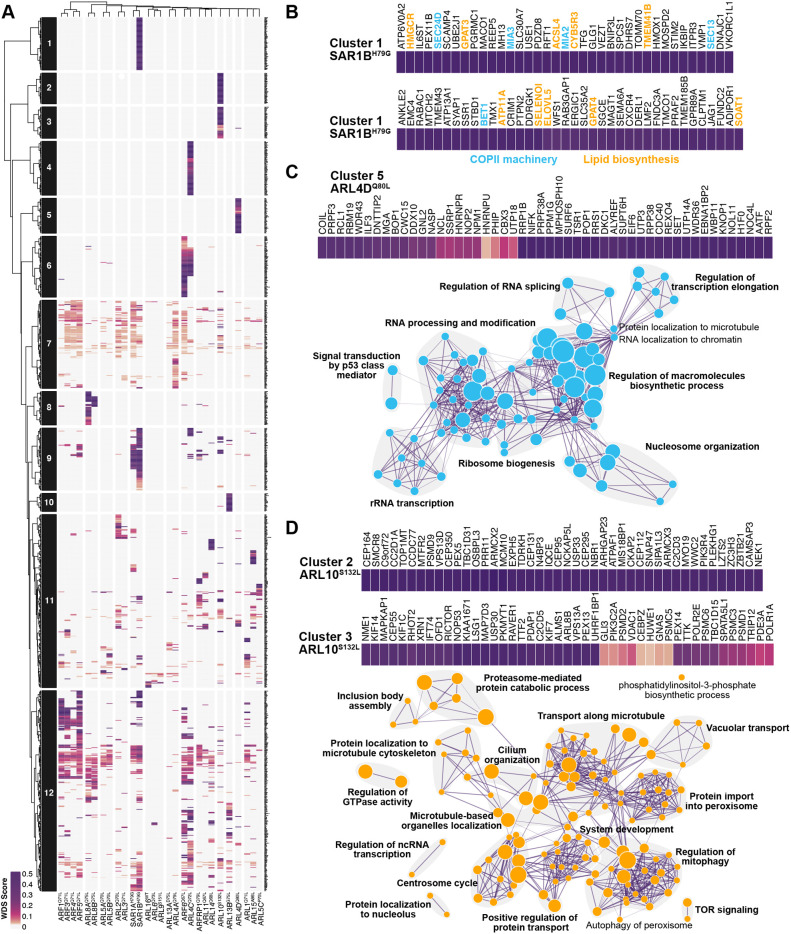
**Clustering of ARF and ARL candidate effectors.** (A) Heatmap of the specificity of interactors of ARFs/ARLs, determined by Pearson correlation. Dark purple signals represent the highest level of bait–prey specificity (highest WDS score). (B) Zoom into cluster 1, which is specific to active SAR1B, reveals the presence of proteins involved in COPII machinery (blue) and lipid biosynthesis (orange). (C,D) Zoom into cluster 5 (C), which is associated with active ARL4D, or into clusters 2 and 3 (D), which are linked to active ARL10, are shown on the top. The bottom panels represent an enrichment map of overrepresented Gene Ontology ‘biological processes’ for the corresponding clusters. Node size reflects the number of proteins associated with each term, and the length of the edges indicates the interconnectivity between terms. Bold text indicates grouped processes.

A major objective was to take advantage of the BioID data of active ARFs and ARLs to identify effector proteins. To determine the specificity of the interactors identified in individual datasets, we generated a computational WD summed (WDS) score, which corresponds to the addition of the WD score for each cell line. The WD score is a proteomic metric that takes into account the spectral counts, the frequency, the reproducibility and the variation (Sowa et al., 2009). We then conducted clustering to highlight the distinctiveness within the set of preys identified with a particular bait (Fig. 3A). The reliability of this approach is demonstrated by the clustering of baits with their closest relatives that share similar effectors. For instance, classical ARFs, except for ARF6, clustered together, as did ARL5A and ARL5B, ARL8A and ARL8B, and SAR1A and SAR1B (Fig. 3A). To further validate this method, we examined known interactions. For instance, the highest WDS score was associated with TBCD in the BioID of ARL2 in HeLa cells, and the highest WDS score in the ARL1 dataset was for the GRIP domain golgins [GOLGA1 (also known as Golgin97) and GOLGA4] (Table S8), all of which are established interactions.

Clustering analyses revealed several distinct clusters. Cluster 1 was linked with SAR1B (Fig. 3B). SAR1B mutations, unlike those in SAR1A, are implicated in chylomicron retention disease, also known as Anderson disease, resulting in deficient chylomicron secretion and fat malabsorption (Jones et al., 2003; Shoulders et al., 2004). In Caco-2/15 cells, SAR1B silencing increased lipophagy, whereas SAR1A silencing resulted in the opposite phenotype (Sane et al., 2019), highlighting the functional differences between these SAR1 paralogs. These discrepancies are mirrored by their differential proximity interactomes. Notably, components of the COPII machinery, such as SEC24D, involved in cargo recognition, and MIA2, involved in large cargo secretion from the ER-like chylomicron (Santos et al., 2016), were uniquely associated with SAR1B. Additionally, certain preys exclusively passed our stringent BioID thresholding in the SAR1B interactome, including BET1, a v-SNARE required for ER-to-Golgi transport (Mossessova et al., 2003), and MIA3, which is involved in collagen secretion from the ER (Saito et al., 2011; Raote et al., 2020). Moreover, several proteins identified in cluster 1 possess a transmembrane domain, with some involved in lipid metabolism (Fig. 3B). Together, these preys highlighted in the SAR1B dataset delineate avenues for further exploration in the machinery and cargos of SAR1B-associated COPII vesicles.

Pathway enrichment analyses of individual clusters revealed previously unknown localizations and functions for several ARLs. In particular, cluster 5 exhibits enrichment in preys specifically identified with ARL4D, recognized for its involvement in multiple cellular processes such as actin remodeling (Li et al., 2007), neurite formation (Yamauchi et al., 2009), adipogenesis (Yu et al., 2011), microtubule growth (Lin et al., 2020) and immune functions (Geers et al., 2022; Tolksdorf et al., 2018). Overrepresentation analyses of Gene Ontology (GO) ‘biological processes’ (BP) are consistent with ARL4D residency in the nucleus, suggesting potential functions in the nucleolus. Additionally, roles in ribosome biogenesis, nucleosome organization, RNA processing, regulation of RNA splicing, transcription and macromolecule biosynthetic processes are also indicated (Fig. 3C). Clusters 2 and 3 are uniquely associated with ARL10, an uncharacterized protein. Enrichment analysis suggests a role for ARL10 in regulating mitophagy, protein import into peroxisomes, vacuolar transport, microtubule-based transport, cilium organization, the centrosome cycle and TOR signaling (Fig. 3D), consistent with its subcellular localization at the mitochondria and peroxisomes (Fig. 2D). To determine the nucleotide-loaded state of ARL10 in cells, we used the RP-IP-HPLC approach. Following purification, WT ARL10 co-precipitated with both GDP and GTP, suggesting that a significant fraction resides in an activated state (29% GTP; Fig. S6E). In contrast, the constitutively active (S132L) mutant of ARL10 did not bind detectible levels of nucleotides, indicating that the mutation might reduce overall nucleotide affinity as observed with ARF1^Q71L^ and ARL4D^Q80L^. This result was not due to differential quantities of purified protein, as 4-fold more ARL10^S132L^ also lacked any detectable nucleotides (Fig. S6A,E). To further delineate the functional role of ARL10 and obtain insights into its protein–protein interactions, an affinity purification-MS (AP-MS) experiment was performed on WT ARL10–GFP and its active mutant used in the BioID (Table S6). Most preys were identified with both ARL10 forms, albeit with variation in the number of spectral counts. GO analysis of BP revealed enrichment in ‘mitochondrion organization’, ‘mitochondrion transport’, ‘mitophagy’ and ‘peroxisome organization’ for both variants. Additionally, WT ARL10 displayed strong enrichment in processes related to organelle localization, organic acid metabolism, protein localization to peroxisomes and TORC2 signaling (Fig. S5B), suggesting the importance of nucleotide binding for these processes. The term ‘TORC2 signaling’ was enriched in both the cluster 3 from the BioID (Fig. 3D) and the AP-MS results, with the core components RICTOR and MAPKAP1 representing mTORC2. The AP-MS also retrieved all other accessory proteins associated with this complex except for DEPTOR, highlighting the favorable interaction of WT ARL10 with these proteins (Fig. S5C). Co-immunoprecipitation confirmed this observation, showing that WT ARL10 exhibits a stronger interaction with RICTOR than the active form (Fig. S5D).

**Fig. 4.**
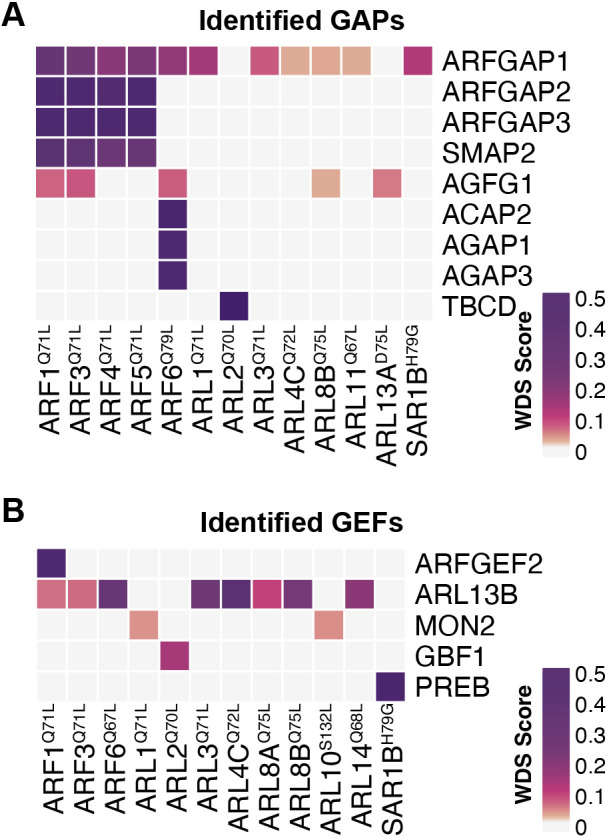
**Mapping of ARF regulators.** Heatmaps illustrate the specificity of GAPs (A) and GEFs (B), as identified through BioID. Dark purple indicates the highest level of bait–prey specificity, as determined by the WDS score.

We also found regulators of the nucleotide-cycling activity of ARFs and ARLs in our BioID datasets. The constitutively active mutants selected for proteomics were predicted to associate preferentially with GAPs, being locked in GTP-loaded states. Interestingly, our data revealed an abundance of GAPs and GEFs in the datasets (Fig. 4). In line with previous findings, ARFGAP1, ARFGAP2 and ARFGAP3 were proximal to the classical ARF1, ARF2, ARF4 and ARF5. Although ARFGAP2 and ARFGAP3 exhibited specificity, ARFGAP1 displayed a degree of promiscuity with several additional GTPases. AGFG1 was identified with class I classical ARFs (i.e. ARF1 and ARF3), the class III ARF (ARF6), as well as ARL8B and ARL13A. Furthermore, ARF6 was proximal to ACAP2, AGAP1 and AGAP3. In comparison to GAPs, the identified GEFs showed narrower specificity: ARFGEF2 was in the BioID of ARF1 and GBF1 was identified with ARL2. Additionally, the GEF-like protein MON2 was identified with ARL1 and ARL10. Notably, ARL13B, a known GEF for ARL3, was identified in numerous ARF datasets. Collectively, our findings unveil several previously unknown interactions among ARFs/ARLs and potential effectors, GEFs and GAPs. Future investigations should aim to further understand their biological significance.

### The understudied ARL14 is a robust activator of PLD1

**Fig. 5.**
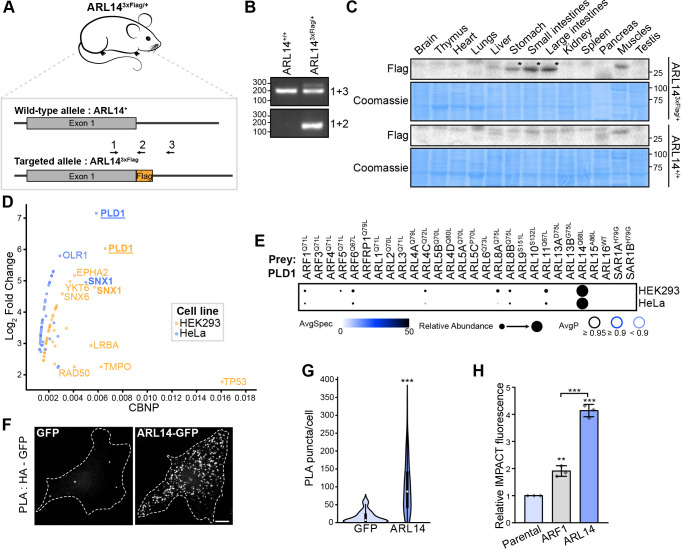
**Characterization of ARL14, a PLD1 activator.** (A) Schematic representation of the murine *Arl14* coding region (one exon), targeted for genome editing. The genotyping strategy is indicated with arrows representing the different primers used in B. (B) Genotyping results of ARL14^+/+^ (WT) or ARL14^3×Flag/+^ knock-in mice. (C) Western blots for proteins isolated from organs of ARL14^+/+^ or ARL14^3×Flag/+^ knock-in mice at age postnatal day 65. The asterisks (*) indicate the bands of interest. Coomassie staining was used as a loading control (*n*=3). (D) The graph represents preys identified in ARL14 BioID, showing the normalized spectral counts (CBNP) on the *x*-axis and the log_2_ fold change against the mean of the negative controls on the *y*-axis. (E) Dotplot showing proximity interactions of PLD1 with ARF GTPases. Darker circle colors represent higher spectral counts, circle sizes denote relative abundance, and circle outlines correspond to the indicated average probability (AvgP). AvgSpec, average spectral count. (F) Proximity ligation assay (PLA) on Flp-In HeLa cells expressing HA–PLD1 and ARL14–GFP. Cells expressing HA–PLD1 and GFP alone were used as a control. F-actin staining (not shown) was used to delineate the cell outline. Scale bar: 10 µm. (G) The violin plot illustrates the number of PLA puncta per cell from the experiment presented in F (total of 75 cells per condition; *n*=3). *P*-value was calculated by two-tailed unpaired Student’s *t*-test; ***P<0.0001. (H) Measurements of PLD activity using IMPACT on ARF1^WT^ and ARL14^WT^. The graph shows the relative IMPACT fluorescence (mean±s.d.) quantified by flow cytometry (*n*=3). *P*-values were calculated by one-way ANOVA, followed by Bonferroni's test; **P<0.001; ***P<0.0001.

Many members of the ARF family have remained relatively unexplored, with ARL14 backed only with a few reports for a role in lung cancer progression (Guo et al., 2019; Zhang et al., 2021). According to single-cell mRNA expression data archived in the Human Protein Atlas, ARL14 expression is reported to be high in the gallbladder, stomach and intestines, with lower levels observed in the urinary bladder and pancreas (Uhlen et al., 2015). In the absence of suitable antibodies, we opted to validate the expression patterns of the ARL14 protein *in vivo* in mice by tagging the endogenous C-terminus of ARL14 with a 3×Flag epitope using the ‘improved genome-editing via oviductal nucleic acid delivery’ (*i*-GONAD) method (Gurumurthy et al., 2019). The mice were validated through genomic locus sequencing and two genotyping strategies (Fig. 5A,B). We extracted proteins from a range of organs from adult WT or transgenic ARL14^+/3×Flag^ mice, and western blotting analyses revealed exclusive expression of ARL14 in the stomach, small intestine and large intestine (Fig. 5C).

**Fig. 6.**
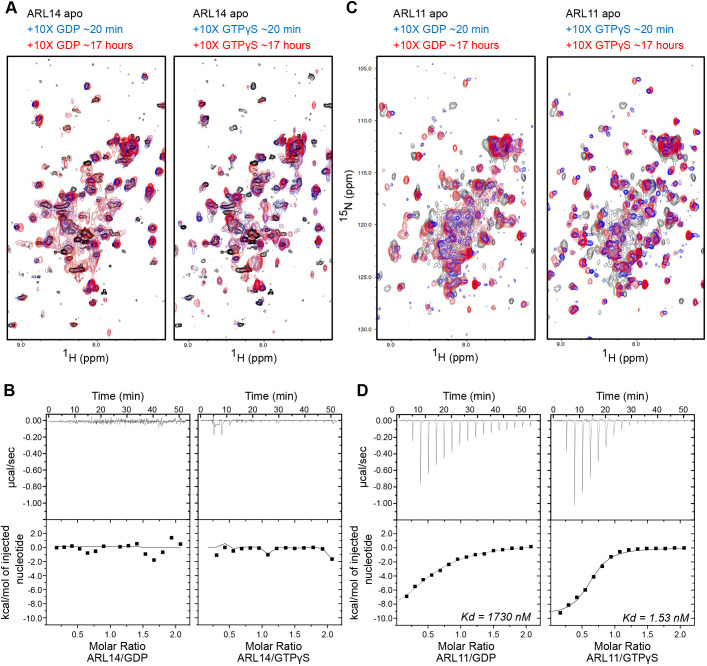
**ARL14 has poor affinity for nucleotides.** (A,C) NMR spectra of purified ARL14 (A) or ARL11 (C) incubated with GDP or GTPγS. (B,D) Isothermal titration calorimetry analysis of ARL14 (B) or ARL11 (D) with GDP or GTPγS. K_d_, dissociation constant.

There are no biochemical data describing ARL14 function. To understand its nucleotide cycling, we first used IP-RP-HPLC of the WT protein and active (Q68L) mutant, which revealed no discernable nucleotide binding (Fig. S6F). As low quantities of ARL14 could be expressed and purified on beads (Fig. S6A), we attempted to study its cycling *in vitro*. Bacterially purified ARL14 was noticeably unstable and ^1^H-^15^N heteronuclear single quantum coherence (HSQC) nuclear magnetic resonance (NMR) spectra presented few robust or well-dispersed peaks characteristic of folded, nucleotide-bound small GTPases (Smith and Ikura, 2014; Killoran and Smith, 2019). This could not be improved by addition of GDP or a non-hydrolyzable analog of GTP (GTPγS; Fig. 6A). We corroborated these results by measuring the affinity of ARL14 to GDP and GTPγS using isothermal titration calorimetry (ITC), which confirmed that ARL14 is unable to bind either nucleotide (Fig. 6B). These data validate the RP-IP-HPLC assay and suggest that ARL14 is unlikely to bind guanine nucleotides. We considered if this was a general property of the subfamily, which includes ARL4A, ARL4C and ARL4D and the closest relative ARL11 (Colicelli, 2004). Purified ^15^N-ARL11 also presented poorly ordered resonances in HSQC spectra, but in contrast to ARL14, there was a significant improvement in chemical shift dispersion and lineshape upon addition of GTPγS, and to a lesser extent GDP (Fig. 6C). ITC experiments validated binding of ARL11 to GTPγS (K_d_=1.53 nM) and GDP (K_d_=1730 nM), corroborating the NMR results and establishing that ARL11 has an approximately 1000-fold higher affinity for GTP over GDP (Fig. 6D). This is a major deviation from the behavior of classical RAS GTPases, which maintain equal affinity to the two nucleotides, demonstrating significant biochemical divergence of some ARF/ARL GTPases that will undoubtedly impact function.

To investigate the molecular basis of ARL14 signaling, we opted to conduct experiments using only the WT variant to accurately reflect its nucleotide cycling *in cellulo*. We analyzed our BioID data to identify potential interacting partners. Among the notable proteins identified in the ARL14 interactome was PLD1. Our data exhibited significant spectral counts and the highest log_2_ fold change compared to the mean of the negative controls for PLD1 in both HEK293 and HeLa cells (Fig. 5D). PLD1 functions by catalyzing the hydrolysis of its substrate, phosphatidylcholine, a phospholipid found in cellular membranes, yielding choline and phosphatidic acid (PA) products. PA is a cone-shaped signaling lipid that facilitates negative curvature of membranes and also interacts with PA-binding proteins required for membrane fusion and fission (Zhukovsky et al., 2019; Roth, 1999). Notably, classical interactors of PLD1 include ARF1 and ARF6, which act as activators, establishing PLD1 as an effector of these GTPases (Hammond et al., 1995; Frohman and Morris, 1999). Although interactions between ARF1 and ARF6 and PLD1 were detected by BioID, the significantly higher PLD1 spectral counts observed in the ARL14 BioID experiments suggest the potential role of ARL14 in activating PLD1 (Fig. 5E). Among the 28 ARFs/ARLs tested, low spectral counts for PLD1 were observed in the BioID studies of ARL4C, ARL8A, ARL8B and ARL11, similar to those in the ARF1 and ARF6 experiments, implying a robust interaction with ARL14. To validate this interaction, we conducted a proximity ligation assay (PLA) in HeLa cells overexpressing WT ARL14–GFP and HA–PLD1, which confirmed that PLD1 is proximal to ARL14 (Fig. 5F,G). To assess whether ARL14 could activate PLD1, we used a click chemistry method named IMPACT, which stands for ‘imaging PLD activity with clickable alcohols via transphosphatidylation’ (Bumpus and Baskin, 2017; Bumpus et al., 2020). IMPACT involves two steps: (1) production of a clickable lipid via PLD-mediated transphosphatidylation of phosphatidylcholine with an exogenous alcohol (3-azidopropanol) and (2) tagging the clickable lipid with a fluorophore (BCN-BODIPY) by a click chemistry reaction. This experiment was performed in HeLa cells overexpressing either mScarlet, ARF1^WT^–mScarlet, or ARL14^WT^–mScarlet, and the fluorescent lipid product of the IMPACT reaction was quantified by flow cytometry (Fig. 5H). This *in cellulo* approach demonstrated that ARL14 was a stronger PLD1 activator than ARF1, with a 4.5-fold and 2-fold increase in fluorescence, respectively, compared to that of the mScarlet control. These findings reveal ARL14 as a robust activator of PLD1.

### ARL14 is involved in ESCPE-1-mediated trafficking

**Fig. 7.**
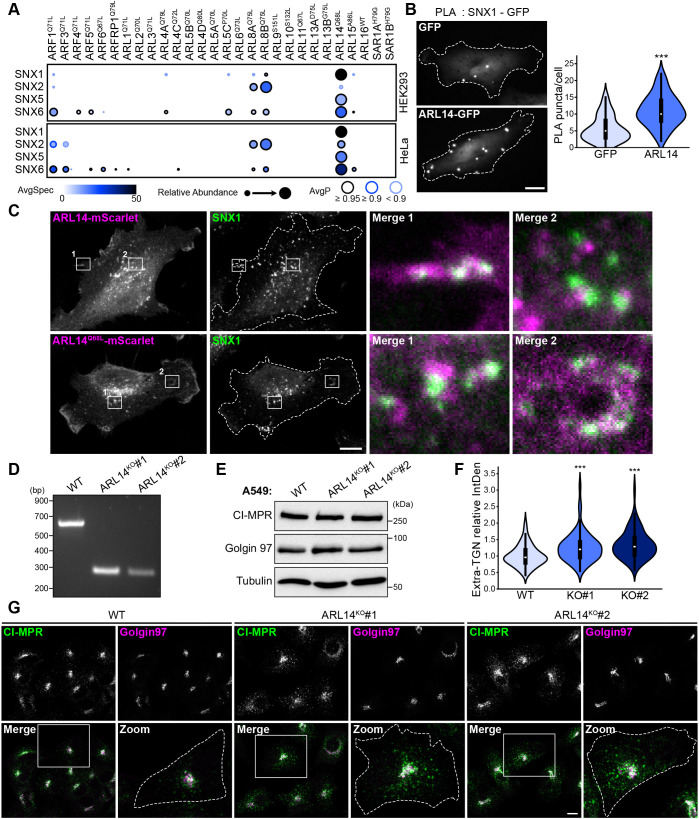
**ARL14 is involved in CI-MPR retrograde trafficking through ESCPE-1.** (A) Dotplots showing the proximity interactions of the ESCPE-1 complex components (SNX1, SNX2, SNX5 and SNX6) with ARF GTPases. Darker circle colors represent higher spectral counts, circle sizes represent relative abundance, and circle outlines correspond to the indicated average probability (AvgP). AvgSpec, average spectral count. (B) Proximity ligation assay (PLA) was conducted on endogenous SNX1 and overexpressed GFP versus ARL14^WT^–GFP in Flp-In HeLa cells. Scale bar: 10 µm. The violin plot shows the quantification of the number of PLA puncta per cell (total of 90 cells per condition; *n*=3). *P*-value was calculated by two-tailed unpaired Student’s *t*-test; ***P<0.0001. (C) SNX1 immunostaining in Flp-In HeLa cells expressing ARL14^WT^–mScarlet or the active variant (Q68L). Scale bar: 10 µm. Images are representative of *n*=3 independent experiments. (D) Validation of *ARL14* knockout (KO) in A549 cells by PCR. The genotyping strategy predicts a band of 610 bp in wild-type (WT) cells and of 280 bp in KO cells. (E) Western blot of total proteins extracted from WT or *ARL14* KO A549 cells demonstrating no alterations in the expression level of CI-MPR or Golgin97. (F) Violin plot shows the quantification of the integrated density (IntDen) outside the TGN relative to that in the control, for each condition presented in G (total of 120 cells per condition; *n*=3). *P*-values were calculated by one-way ANOVA, followed by Bonferroni's test; ***P<0.0001. (G) Co-immunostaining was performed using anti-Golgin97 (TGN marker) and anti-CI-MPR in WT or *ARL14* KO A549 cells. Dotted lines delineate cell outlines, which were defined using F-actin staining (not shown). Scale bar: 10 µm.

In addition to PLD1, the endosomal SNX-BAR sorting complex for promoting exit-1 (ESCPE-1) complex, which consists of dimers of SNX1 or SNX2 with SNX5 or SNX6, also emerged as a highly specific proximity interactor of ARL14 (Fig. 7A). SNX1 was identified among the preys with high spectral counts and elevated log_2_ fold change compared to the mean of the negative controls in both HEK293 and HeLa cells (Fig. 5D). To corroborate the BioID data, PLA was conducted on Flp-In T-Rex HeLa cells overexpressing WT ARL14–GFP. These experiments demonstrated that endogenous SNX1 (Fig. 7B) and SNX6 (Fig. S7A) are proximal to ARL14, indicating a possible association with the ESCPE-1 complex.

To delineate the subcellular localization of the ARL14 and ESCPE-1 complex, Flp-In HeLa cells expressing WT or the potentially active mutant (Q68L) of ARL14–mScarlet were fixed and subjected to staining for SNX1 or SNX6. Given the pivotal role of SNX-BAR proteins in both endosome-to-plasma membrane recycling and endosome-to-*trans*-Golgi network (TGN) retrieval (Simonetti et al., 2023), we predicted that ARL14 and SNX would exhibit colocalization in at least one of these sites. These experiments revealed that both WT and Q68L ARL14–mScarlet were partially soluble in the cytoplasm, with some enrichment in punctate structures reminiscent of endosomes, as well as at the plasma membrane (Fig. 7C; Fig. S7B). Puncta positive for either WT or Q68L ARL14–mScarlet frequently appeared adjacent to SNX1 or SNX6 puncta, as depicted in the magnified images (Fig. 7C; Fig. S7B). Furthermore, we observed an accumulation of SNX1 and SNX6 puncta along domains of the plasma membrane enriched for WT or Q68L ARL14–mScarlet (Fig. 7C; Fig. S7B). These findings (1) suggest that the Q68L mutation, introduced to constitutively activate ARL14 for the BioID, did not alter ARL14 localization and (2) indicate the proximity of ARL14 to the ESCPE-1 complex.

In addition to its roles in phospholipid recognition and membrane tubulation, ESCPE-1 also acts as a recognition and sorting module for a specific subset of transmembrane proteins transiting in the endosomes (Simonetti et al., 2019). One well-studied cargo of ESCPE-1 is the cation-independent mannose-6-phosphate receptor (CI-MPR, also known as IGF2R) (Fig. S7C). Depletion of SNX5 and SNX6 was previously demonstrated to reduce CI-MPR retrieval from endosomes to the TGN, resulting in an accumulation of CI-MPR outside the TGN (Kvainickas et al., 2017; Simonetti et al., 2017). We were able to replicate these findings in HeLa cells using pools of small interfering RNAs (siRNAs) targeting *SNX5* and *SNX6* (Fig. S7D,F). To investigate the potential role of ARL14 in ESCPE-1-mediated trafficking, we used CRISPR/Cas9 to generate two *ARL14* knockout cellular models. We worked with HeLa cells, which exhibit low expression of ARL14 but directly link the BioID screens. Additionally, we employed A549 lung cancer cells, previously shown to express ARL14 and in which ARL14 was demonstrated to enhance cell proliferation, migration and invasion (Guo et al., 2019). Efficiency of *ARL14* genomic deletion was validated through genomic DNA sequencing (HeLa, Fig. S7G) and PCR amplifications (A549, Fig. 7D). One clone in HeLa and two independent clones in A549 were validated and selected to further investigate the impact of ARL14 on CI-MPR localization. As the CI-MPR localization assay relies on staining of CI-MPR and Golgin97, we first confirmed that the absence of ARL14 does not lead to variation in the expression levels of CI-MPR or Golgin97 (Fig. 7E). Next, by comparing the localization of CI-MPR in or outside of the TGN marked by Golgin97 in control or ARL14 knockout cells, we demonstrated that ARL14 is crucial for the retrieval of CI-MPR to the TGN (A549, Fig. 7F,G; HeLa, Fig. S7H,I). These findings collectively support a previously unknown functional association between ARL14 and the ESCPE-1 in regulating endosomal trafficking.

## DISCUSSION

The ARF/ARL GTPases regulate a broad array of cellular processes, with dynamic protein–protein interactions occurring in membrane-rich compartments being indispensable. Although classical ARFs have been extensively investigated, the localization and functions of ARL proteins remain less defined. In this study, we systematically elucidated the proximity interactomes of ARFs/ARLs using BioID, a tool for identifying stable or transient protein interactions within their native milieu. Our findings significantly broaden the repertoire of candidate interactors associated with ARF/ARL proteins. These data, available for public access, hold considerable potential for the research community to formulate novel hypotheses. Our investigations represent just the initial step in extracting new insights from this large dataset, particularly regarding the subcellular compartments from which each ARF and ARL exerts its functions, their proximity interactors and new functional implications for the understudied members, such as ARL10 and ARL14.

Our approach has limitations, particularly in our requirement to express tagged proteins, which can lead to the creation of artifacts, hinder some interactions or alter functionality. Additionally, the localization of the GTPases can be skewed to favor one organelle over another. For instance, ARL4D was predominantly localized in the nucleus, despite the report that its localization to the plasma membrane can be stabilized by binding to the chaperone-like protein HYPK (Lin et al., 2022). It remains important to validate BioID interactions using additional methods. Moreover, our analysis of ARFs/ARLs was restricted to HEK293 and HeLa cells, possibly missing unique functions and additional interactors specific to specialized cell types. For example, as reported in this study, ARL14 is predominantly expressed in gastrointestinal cells.

As the endogenous activation mechanisms for most ARF/ARL GTPases are unknown, we performed the BioID screen using mutants that are presumed constitutively active, based on homology to the KRAS^Q61L^ variant. This approach proved valuable in enriching our datasets with previously unreported effectors and regulatory proteins, and in helping obtain organelle-specific functions, although undoubtedly ARF/ARL cycling is important for function completion (Klein et al., 2006). Working with unstudied ARLs raises the caveat of uncertainty regarding the effect of the activating mutations on cycling, likely exacerbated by their weaker affinity for nucleotides compared to that of RAS and RHO GTPases (Jacobs et al., 1999; Linari et al., 1999; Hanzal-Bayer et al., 2005). Indeed, our RP-IP-HPLC approach provides the first ever glimpse at ARF GTPase nucleotide loading in cells, and the data suggest that neither the WT proteins nor ‘activated’ mutants behave in a manner completely analogous to that of classical RAS. Our NMR and ITC experiments begin to elucidate the biochemical properties of a subfamily of ARFs comprising ARL4A, ARL4C, ARL4D, ARL11 and ARL14 (Colicelli, 2004). It appears unlikely that ARL14 is regulated by GDP/GTP cycling as it has no affinity for either nucleotide, whereas ARL11 has an unusually enhanced affinity for GTP. This remains significantly lower than that of classical RAS proteins that bind both GTP and GDP with low picomolar affinity (Ford et al., 2009). NMR spectra did not present the well-dispersed peaks characteristic of RAS and RHO GTPases, and it is probable that ARL11 and ARL14 are purified from bacteria in an apo state. Previous work suggests this is also true of ARL4A and ARL4C (Jacobs et al., 1999), and these results are consistent with our HPLC data. Although ARF proteins are difficult to express and purify, future efforts must fully characterize the biochemical activity of all ARFs/ARLs to allow improved design of constitutively active and inactive variants.

An objective of this study was to attribute potential functions to less-characterized members of the ARF family. Among these, the understudied ARL10 caught our attention due to its localization to mitochondria, mediated by a distinctive transmembrane domain followed by a polybasic region. Both BioID and complementary AP-MS studies emphasized the potential role of ARL10 in mitophagy, mitochondrial fission and various metabolic processes. Furthermore, our experiments indicate an association between ARL10 and the mTORC2 complex. Given the localization of ARL10, it might provide insights into the function and regulation of the small active pool of mTORC2 previously observed at the mitochondria (Ebner et al., 2017). Furthermore, ARL10 localizes to the peroxisomes, an organelle closely interconnected to mitochondria that communicate through small vesicles (Neuspiel et al., 2008). Because of the dual localization of ARL10 and its proximity to proteins involved in mitochondrial dynamics, an attractive hypothesis for future research is that ARL10 might control vesicular traffic between mitochondria and peroxisomes.

While our study was nearing completion, Li et al. (2022) published a miniTurboID interactome of the ARF family, labeling for 15 min and targeting 25 constitutively active members. In contrast to our method of expressing baits in a tetracycline-inducible manner, they employed stable overexpression by infecting HEK293A cells. Based on our experience with RHO proteins, we chose to use an inducible system, to mitigate toxicity or gene expression alterations resulting from stable and uncontrolled overexpression of active GTPases. The Li et al. (2022) study and our work are complementary and demonstrate that short- versus long-term labeling can yield unique insights. Shorter labeling highlights frequently occurring interactions, whereas longer labeling captures more transient interactions, increases the overall number of proximal interactors and reveals interactions dependent on specific cell states (e.g. during specific stages of the cell cycle). We compared the hits from Li et al. (2022) to our HEK293 cell datasets for the 24 common baits and estimate ∼350 shared interactions, leaving nearly 1400 new proximity interactors identified by our BioID. A limitation of the Li et al. (2022) approach is the lower spectral counts observed (due to short labeling time), resulting in very low correlation coefficients among replicates (some as low as 0.4). In our study, all replicates show correlation coefficients above 0.9. Additionally, statistical data are not provided to support the confidence of the reported proximity interactions, whereas we employed rigorous filtering using non-specific baits (BirA*, BirA*–eGFP and BirA*–eGFP–CAAX) and we provide statistical confidence for all our data. In conclusion, we believe that these two studies represent a significant advancement in obtaining an accurate representation of the entire ARF family interactome.

Like us, Li et al. (2022) noted the previously unexplored interaction between PLD1 and ARL14. Although they demonstrated that ARL14 is a PLD1 activator *in vitro*, we used an *in cellulo* approach, with the two methods thereby strengthening the conclusion that ARL14 is a *bona fide* activator of PLD1. Although Li et al. (2022) defined the essential region in PLD1 for ARL14 binding, we conducted functional studies that led to the identification of the ESCPE-1 complex as a potential effector of ARL14. Notably, our experiments demonstrated a reduction in CI-MPR retrieval in the Golgi of ARL14-null A549 and HeLa cells. We identified SNX1, SNX2, SNX5 and SNX6 as relatively specific proximal interactors of ARL14, but only SNX1 was identified in the ARL14 miniTurboID of Li et al. (2022). Based on our findings, we postulate that the ESCPE-1 complex and PLD1 might operate in collaboration to generate endosomes. ARL14 could insert into endosomal membranes, inducing local positive curvature, thereby attracting ESCPE-1 as coats, leading to tubulation and concentration of cargos. Concurrently, ARL14 could recruit and activate PLD1 to produce PA, enhancing membrane curvature for endosome formation. Additionally, our study reveals the restricted expression of ARL14 in gastrointestinal cells of mice. Fully characterizing the role of ARL14 and its interactors PLD1 and ESCPE-1 in specialized cells of the intestines and within the context of lung cancer, where these protein–protein interactions might play crucial roles in secretory functions, represents a biologically relevant future area of investigations.

Overall, our study underscores the potential of our dataset in uncovering previously unknown functions and regulatory mechanisms of ARF and ARL proteins. Our BioID data are accessible on our website (http://prohits-web.lunenfeld.ca), serving as a valuable resource for the research community interested in exploring interactors of ARF and ARL proteins.

## MATERIALS AND METHODS

### Antibodies, plasmids, siRNAs, primers, cDNA and cloning

The antibodies used in this study are detailed in Table S2, plasmids are catalogued in Table S3, siRNAs in Table S4 and primers in Table S5. The NCBI reference sequence numbers of the complementary DNA (cDNA) of ARF/ARL genes used in this study are listed in Table S1. All coding sequences were synthesized as previously described (Patel et al., 2011), with the exception of those for ARL6, ARL9 and ARL16, for which sequences were obtained from NCBI and synthetized with addition of Gateway-compatible AttB sites (Twist Bioscience). The Gateway cloning system (Invitrogen) was used to recombine the cDNA sequences into the entry vector (pDONR221; V600520, Thermo Fisher Scientific). Subsequently, each ARF/ARL cDNA was recombined into the expression vector pcDNA5-pDEST-FRT-BirA*-Flag-CT (Couzens et al., 2013). For certain proteins, alternative destination vectors were used to clone in-frame fluorescent protein coding sequences, namely, pcDNA5-pDEST-FRT-eGFP-CT (Kean et al., 2011) was used for ARL10 and ARL14, and pcDNA5-pDEST-FRT-mScarlet-CT (generated as follows) for ARL14. To construct pcDNA5-pDEST-FRT-mScarlet-CT, the coding sequence of mScarlet was amplified by PCR, with pMaCTag-Z11 (Addgene #120054) as a template DNA, using primers engineered with KpnI/XhoI cloning sites, such that the mScarlet cDNA replaced the eGFP sequence in pcDNA5-pDEST-FRT-eGFP-CT after digestion with KpnI and XhoI and ligation.

### Cell culture and transfections

Flp-In T-Rex HEK293 (Thermo Fisher Scientific), Flp-In T-Rex HeLa (gift from Stephen Taylor, University of Manchester, UK), HEK293T and HeLa (both from American Type Culture Collection) cell lines were maintained at 37°C and 5% CO_2_ and grown in Dulbecco's modified Eagle's medium (DMEM; 319-005-CL, Wisent) supplemented with 10% fetal bovine serum (FBS; F1051, Sigma-Aldrich) and 1% penicillin–streptomycin (P/S) (450-201-EL, Wisent). All cells were tested negative for mycoplasma contamination. Stable cell lines were generated as previously described (Nahle et al., 2022) and were grown in DMEM supplemented with 10% tetracycline-free FBS (081-150, Wisent) and 1% P/S. To induce protein expression, stable cell lines were treated with 1 μg/ml of tetracycline with or without 50 µM biotin (BB0078, Bio Basic) supplementation. siRNA transfections were performed using Dharmafect-1 (GE Healthcare) and 80 nM of the indicated siRNA. Plasmid DNA transfections were performed with Lipofectamine 3000 (L3000001, Thermo Fisher Scientific), according to the manufacturer’s instructions.

### Protein expression and purification

GST-tagged ARL11 (amino acids 1–196) and ARL14 (amino acids 1–192) were expressed and purified from *Escherichia coli* (BL21-DE3-codon+) cells cultured in LB media. For NMR, isotopically labeled proteins were obtained by growing BL21-DE3-codon+ cells in M9 minimal media (50 mM Na_2_HPO4, 22 mM KH_2_PO4, 8.5 mM NaCl, 1% glucose, 1 mM MgSO_4_, 0.1 mM CaCl_2_, 38 µM thiamine, pH 7.4), and for ^15^N-uniformly labeled protein samples, 1 g of [^15^N]NH_4_Cl was added. Cells were grown at 37°C until they reached an optical density of 0.6–0.8, followed by induction with 250 µM of isopropyl-β-D-thiogalactopyranoside (800-050-EG, Wisent). After induction, the temperature was decreased to 18°C and cells were grown overnight. Harvested cells were lysed in a buffer containing 20 mM Tris-HCl pH 7.5, 150 mM NaCl, 10% glycerol, 0.4% NP-40, protease inhibitors (Roche) and 2 mM dithiothreitol (DTT), followed by sonication. Each lysate was clarified by centrifugation and next incubated with glutathione sepharose resin (17-0756-04, Cytiva) at 4°C for 1–2 h. Subsequently, bound proteins were cleaved from the resin, without elution, using thrombin protease (91-035-100, BioPharm). The proteins recovered after cleavage underwent size-exclusion chromatography using a Superdex S75/300 column (GE Healthcare) in a phosphate-based buffer (200 mM sodium phosphate pH 7.4, 15 mM HEPES pH 7.4, 10 mM MgCl_2_, 5 mM KCl, 2 mM DTT).

### HPLC nucleotide analysis

One 15 cm plate of HEK293T cells was transfected with the indicated plasmids using Opti-MEM (31985062, Thermo Fisher Scientific) and polyethylenimine (23966-1, Polysciences), followed by harvest after 48 h. To isolate GFP-tagged GTPases, the cells were suspended in lysate buffer (20 mM Tris-HCl pH 7.5, 150 mM NaCl, 5 mM MgCl_2_, 10% glycerol, 1% NP-40, 1% Triton X-100, 1 mM DTT and protease inhibitors), then incubated on ice for 15 min. Each lysate was then cleared at 14,000 ***g*** for 10 min at 4°C, and the cleared lysate was incubated with N-hydroxysuccinimide beads (GE17-0906-01, Cytiva) pre-conjugated with anti-GFP nanobodies (purified in house; Schellenberg et al., 2018) for 30 min. Beads underwent a single buffer wash step to minimize the loss of weakly bound nucleotides using the following wash buffer: 20 mM Tris-HCl pH 7.5, 150 mM NaCl, 5 mM MgCl_2_ and 1 mM DTT. Following this, the beads were resuspended in 200 µl of wash buffer and boiled at 95°C for 6 min, followed by centrifugation at 14,000 ***g*** for 10 min. The supernatant, containing released nucleotides, was filtered through pre-washed 0.22 µm PVDF centrifugal filters (UFC30GV25, Millipore). The flow through was used for HPLC analysis, conducted using an Agilent 1100 Series HPLC and C18 column (Eclipse XDB-C18 5 μm, 4.6×150 mm, Agilent). The running phase was prepared in 500 ml of a solution containing 6.29 g KH_2_PO_4_, 1.49 g tetrabutylammonium bromide, and 6.8% acetonitrile in water. Each sample was run in the mobile phase at a flow rate of 0.95 ml/min for 9 min at 25°C, with absorbance detected at 252 and 254 nm.

### BioID and MS injections

BioID experiments were performed as previously described (Bagci et al., 2020), with additional protocols for BioID and MS injection further detailed in Nahle et al. (2022). Briefly, cells were lysed using RIPA lysis buffer (1% NP-40, 50 mM Tris-HCl pH 7.4, 0.1% SDS, 150 mM NaCl, 0.5% sodium deoxycholate, 1 mM EDTA pH 8), followed by sonication. Biotinylated proteins were isolated from cleared lysates by incubating with 70 µl of streptavidin beads (17-5113-01, GE Healthcare) at 4°C for 3 h. Following successive washes with lysis buffer and ammonium bicarbonate (AB0032, Bio Basic), on-bead digestion was performed overnight with 1 ng/µl of trypsin (T6567, Sigma-Aldrich), followed by an additional 2 h incubation with 1 ng/µl of fresh trypsin. The digested peptides were recovered from the mix containing beads by washes with water. Tryptic digestion was terminated by adding formic acid (94318, Sigma-Aldrich) to a final concentration of 5% to end. Peptides were then dried down in a SpeedVac vacuum concentrator, resuspended in 15 µl of 5% formic acid and stored at −80°C. Samples generated from HEK293 cells were analyzed with an LTQ-Orbitrap Velos mass spectrometer (Thermo Fisher Scientific) and samples from HeLa cells were analyzed with a Q Exactive mass spectrometer (Thermo Fisher Scientific). Peptide samples were loaded into a 75 μm (internal diameter)×150 mm Self-Pack C18 column installed on the Easy-nLC 1000 system (Proxeon Biosystems). Elution of peptides was performed with a two-slope gradient at a flow rate of 250 nl/min, from 2 to 35% in 100 min and then from 35 to 80% in 10 min, transferring from buffer A (0.2% formic acid) to buffer B (90% acetonitrile, 0.2% formic acid). Ionization of peptides was achieved using a Nanospray Flex Ion Source (ES071, Thermo Fisher Scientific) on both instruments. Acquisition in the Orbitrap was made with a full-scan MS survey scanning (m/z 360–2000) in profile mode with a resolution of 60,000, an automatic gain control (AGC) target at 1E6 and a maximum ion time of 100 ms for both machines. Nanospray and S-lens voltages were set to 1.3–1.8 kV and 50 V, respectively. Capillary temperature was set to 250°C. On the LTQ Orbitrap Velos, the 11 most intense peptide ions were fragmented by collision-induced dissociation fragmentation and MS/MS spectra were acquired in the linear ion trap with an AGC target at 1E4 and a maximum ion time of 100 ms. The MS/MS parameters were: normalized collision energy, 35 V; activation q, 0.25; activation time, 10 ms; and target ions already selected for MS/MS were dynamically excluded for 30 s. For the Q Exactive, the 16 most intense peptide ions were fragmented by higher-energy collisional fragmentation and MS/MS spectra were acquired in the Orbitrap with a resolution of 17,500, an AGC target at 1E5 and a maximum ion time of 50 ms. The MS/MS parameters were: normalized collision energy at 27; and target ions already selected for MS/MS were dynamically excluded for 7 s.

### MS data analyses

The raw MS files underwent analysis using the X! Tandem (Craig and Beavis, 2004) and Mascot search engines through the iProphet pipeline (Shteynberg et al., 2011) integrated in the Prohits analysis suite (Liu et al., 2010). The searches were performed using the Human RefSeq protein sequence database (2017 version) supplemented with ‘common contaminants’ from the Max Planck Institute (http://maxquant.org/downloads.htm), the Global Proteome Machine (GPM; http://www.thegpm.org/crap/index.html), and along with decoy sequences. Mascot parameters were configured with strict trypsin specificity (allowing for two missed cleavage sites) and oxidation (M) and deamidation (NQ) considered as variable modifications. The mass tolerances for precursor and fragment ions were set to 10 ppm and 0.6 Da, respectively, with peptide charges of +2, +3 and +4 being considered. The resulting X! Tandem and Mascot search results were individually processed by PeptideProphet (Keller et al., 2002) and peptides were assembled into proteins using parsimony rules first described in ProteinProphet (Nesvizhskii et al., 2003) using the Trans-Proteomic Pipeline (TPP; http://www.tppms.org/). TPP settings were as follows: -p 0.05 -x20 -PPM –d“DECOY”, iprophet options: pPRIME and PeptideProphet: pP.

Sample reproducibility among biological replicates was assessed in R (https://www.r-project.org/) by performing multidimensional scaling with the limma package (Ritchie et al., 2015) and Spearman correlations, with the stats package (https://www.r-project.org/) using spectral counts of identified preys. We discarded and reacquired biological replicates displaying Spearman correlations lower than 0.9 and/or those that clustered poorly among biological replicates on a multidimensional scaling plot.

### Interaction scoring

To calculate statistics on the interactions, we used SAINTexpress (Teo et al., 2014) (version 3.6.1) on proteins with iProphet protein probability ≥0.9 and unique peptides ≥2. Proteomics datasets (ARF BioID screens in HEK293 and HeLa cells) were compared separately against their respective negative controls. These controls comprised pulldowns performed on both cell lines expressing either the eGFP–BirA*–Flag vector or the empty vector (BirA*–Flag alone), each in biological duplicates. SAINT analyses were performed without control or bait compression. To infer high-confidence interacting proteins for ARFs and ARLs, we applied, in addition to the SAINT average probability (AvgP) ≥0.95 threshold (more stringent than a Bayesian false discovery rate ≤0.01 threshold), a combination of two additional filters: the average spectral count (AvgSpec) and the CAAX ratio (Preys AvgSpec/CAAX AvgSpec) for the preys. For each cell line, the cutpoints of both additional filters were estimated by cumulative distribution (CDA) and receiver operating characteristic (ROC) sensitivity analyses. After identifying physical recall interactions for ARFs and ARLs, as reported by the human BioGrid database (v4.4.210) (Chatr-Aryamontri et al., 2017), we performed CDA and ROC sensitivity analysis in R with the mltools (https://github.com/ben519/mltools) and cutpointr (Thiele and Hirschfeld, 2021) packages, respectively. To gain precision, we limited these analyses to ARF1, ARF6, SAR1A and SAR1B, which have been, up to this date, the most characterized proteins of the ARF family. From the results of these approaches, we reasoned that AvgSpec thresholds of 4.5 and 6, with a CAAX ratio of 1.7, were the most appropriate for the HEK293 and HeLa datasets, respectively. Unfiltered contaminants, such as keratin, BirA*, carboxylases and β-galactosidase were manually removed. To assess the specificity of each interaction among the ARF interactomes, we calculated for each cell line, their WD score by using the SMAD R package (Sowa et al., 2009). Next, we pooled the interaction specificities of both cell lines by summing their respective WD scores, giving us a ‘WD summed score’ that we referred to as WDS score. The benefit of this metric is to increase our capacity to identify ARF/ARL interactors of high confidence, either from interactions shared by both cell lines or from unique cell line interactions. We then exploited this WDS score metric by building a heatmap of ARF and ARL effectors with the R complexheatmap package (Gu et al., 2016). To do so, we selected potential ARF and ARL effectors by removing GAPs and GEFs from filtered ARF/ARL interactions. Then, we calculated Canberra distances between filtered preys and Pearson correlations between baits, followed by using the Ward.D method (Murtagh and Legendre, 2014) to extract 16 clusters of preys and 13 clusters of baits. The optimal number of clusters was determined by using the silhouette method from the factoextra R package (http://www.sthda.com/english/rpkgs/factoextra). Similarly, heatmaps of GAPs and GEFs were also created without cluster extraction. For each of the clusters of ARF and ARL effectors, we performed an over-representation analysis of GO BP in R using the gprofiler2 package (Kolberg et al., 2020). The complete lists of interactions with scores and annotations are available online at http://prohits-web.lunenfeld.ca.

### Bioinformatic and network analyses

A proportional area chart was created using the ggplot2 package (Wickham, 2016; https://ggplot2.tidyverse.org) in R. Analysis of the subcellular localization of each ARF/ARL was executed separately on the Cell Map website (https://cell-map.org/), with the results filtered to the top three subcellular localizations with the shortest distances. Graphical representations of protein–protein networks were generated using Cytoscape (Shannon et al., 2003) (version 3.9.1). The illustration of the subcellular localizations of ARFs/ARLs was inspired by the animal cell drawing from the SwissBioPics website (https://www.swissbiopics.org/). Dotplot representations of interactions were generated at the ProHits-viz website (https://prohits-viz.org/). Enrichment maps of GO BP were generated in Cytoscape with the EnrichmentMap app (v 3.3.4) (Merico et al., 2010) by loading overrepresented GO BP estimated with false discovery rate correction by gProfiler (Raudvere et al., 2019). The complexity-based normalization relative to the total identified proteins (CBNP) values were calculated as previously described (Cozzolino et al., 2020).

### Immunofluorescence staining, imaging and analyses

Cells were fixed with 3.7% formaldehyde in CSK buffer (100 mM NaCl, 300 mM sucrose, 3 mM MgCl_2_, 10 mM PIPES, pH 6.8) for 15 min, followed by permeabilization with 0.2% Triton X-100 in PBS for 5 min. Subsequently, cells were incubated with primary antibodies in the wash buffer (1% BSA and 0.1% Triton X-100 in TBS), washed with wash buffer, and incubated for 30 min with the appropriate secondary antibody. Where indicated, Hoechst 33342 (H3570, Invitrogen), Alexa Fluor-conjugated phalloidin and/or streptavidin were simultaneously incubated with secondary antibodies. Afterward, cells were washed with the wash buffer and then with water. Coverslips were mounted with Mowiol. Images were acquired with a Carl Zeiss LSM700 laser scanning confocal microscope (Carl Zeiss MicroImaging, Jena, Germany) equipped with a plan-apochromat 63×/1.4 numerical aperture objective and operated with ZenBlack 2009. FIJI (Schindelin et al., 2012) (v2.0.0-rc-69/1.52 k) was used for analyses. The fluorescence intensity of CI-MPR was quantified by measuring CI-MPR integrated density across the whole cell, excluding the TGN delineated by the Golgin97 staining.

### Co-immunoprecipitation and AP-MS

To enrich for ARL10-associated protein complexes, Flp-In HeLa ARL10–GFP cells were lysed in digitonin buffer (50 mM HEPES pH 7.4, 120 mM NaCl, 0.5% digitonin, 0.25 mM NaVO_3_, 1 mM PMSF, 10 mM NaF) supplemented with EDTA-free protease inhibitor cocktail (4693132001, Sigma-Aldrich). 1 mg of cleared cell lysate was incubated with 10 µl of GFP-selector affinity resin developed by NanoTag Biotechnologies (N0310, Synaptic Systems) for 2 h at 4°C. Beads were washed three times with lysis buffer, resuspended in 30 µl of Laemmli buffer (5% SDS, 0.1 mM Tris pH 6.8, 140 mM β-mercaptoethanol, 25% glycerol) and denatured for 5 min at 95°C for western blotting.

For AP-MS of the ARL10–GFP-associated proteins, the protocol above was followed with the exception that after the three washes of the pulled down GFP-selector beads with lysis buffer, two additional washes with 50 mM HEPES/120 mM NaCl were performed to remove detergents. Samples were then reduced with 9 mM DTT at 37°C for 30 min and, after cooling for 5 min, alkylated with 17 mM iodoacetamide at room temperature for 30 min in the dark. Prior to protein digestion, a protein aggregation capture using hydroxyl beads (ReSyn Biosciences) was performed to remove SDS and glycerol. The proteins were digested on-bead with trypsin (Promega) in a 1:20 enzyme:protein ratio in 50 mM ammonium bicarbonate, performed overnight at 37°C. For each sample, the supernatant was transferred into a new sample tube and dried with a vacuum centrifuge (Eppendorf). Samples were reconstituted under agitation for 15 min in 11 µl of 2% acetonitrile and 1% formic acid and loaded into a 75 μm×150 mm Self-Pack C18 column installed in the Easy-nLC II system (Proxeon Biosystems). Peptides were eluted with a two-slope gradient at a flow rate of 250 nl/min. Solvent B was first increased from 2 to 35% in 120 min and then from 35 to 85% in 20 min. The HPLC system was coupled to an Orbitrap Fusion mass spectrometer (Thermo Fisher Scientific) through a Nanospray Flex Ion Source. Nanospray and S-lens voltages were set to 1.3–1.7 kV and 50 V, respectively. The capillary temperature was set to 225°C. Full-scan MS survey spectra (m/z 360–1560) in profile mode were acquired in the Orbitrap with a resolution of 120,000 with a target value at 1E6. The 25 most intense peptide ions were fragmented in the higher-energy collisional dissociation cell and analyzed in the linear ion trap with a target value at 1E4 and a normalized collision energy at 29 V. Target ions selected for fragmentation were dynamically excluded for 20 s after two MS2 events.

### NMR sample preparation and data acquisition

Uniformly-labeled [^15^N]ARL11 or [^15^N]ARL14 were diluted to 100–200 µM in phosphate buffer on ice. GDP or GTPγS nucleotides were added in 10-fold molar excess to proteins. The final sample solution contained 100–200 µM of protein, 10-fold excess of nucleotide and 10% D_2_O. Samples were centrifuged at 4°C to remove possible precipitation and transferred to a 3 mm NMR sample tube. One dimension ^1^H and two-dimension ^1^H-^15^N BEST-HSQC (band-selective excitation short-transient-HSQC) spectra were acquired using the ZGESGP and B_HSQCETF3GPSI pulse programs, respectively, and recorded on a 600 MHz Bruker Avance III magnet equipped with a tunable ^1^H-^19^F 5 mm Versatile Quadruple Resonance CryoProbe (QCI-P) at 298 K after short (∼10 min) or long (∼17 h) incubation periods with nucleotide. Data were processed using NMRFx Analyst (Norris et al., 2016) and visualized with NMRViewJ (Johnson and Blevins, 1994).

### ITC

The affinity of ARL11 or ARL14 to nucleotides was measured using a MicroCal iTC200 instrument (Malvern). Nucleotide stock solutions were diluted using filtered and degassed phosphate-based buffer (as used for size exclusion chromatography of ARL11 and ARL14). Experiments were carried out at 20°C. Data were processed and visualized using Origin 7 (MicroCal).

### Animal experiments

Mice (*Mus musculus*) were housed in a specific pathogen-free facility, and all experiments were approved by the Animal Care Committee of the Montreal Clinical Research Institute (protocol 2021-04 MK), in compliance with the Canadian Council of Animal Care guidelines. ARL14^3×Flag^ knock-in alleles were generated by CRISPR/Cas9-mediated gene editing via the *i*-GONAD procedure (Gurumurthy et al., 2019). Briefly, a Cas9–ribonucleoprotein mix, containing 6 μM Cas9 protein (S.p. HiFi Cas9 Nuclease V3, 1081061, Integrated DNA Technologies), 30 μM gRNA and the 30 μM ssDNA (coding for 3× Flag), was delivered by microcapillary injection into ampulla of 6- to 10-week-old CD-1 pregnant females at 0.7 days post conception, followed by electroporation of the oviduct. Mice that were born were genotyped by PCR, followed by Sanger sequencing, to verify correct targeting. To select gRNA, we used the IDT tool at https://www.idtdna.com/site/order/designtool/index/CRISPR_CUSTOM. All sequences used are described in Table S5.

For organ collection, male mice of 65 days of age were euthanized. Organs were dissected, flash frozen and kept at −80°C. Each indicated organ was grinded in liquid nitrogen, with an additional shredding step for muscles, and proteins were then extracted with RIPA lysis buffer supplemented with inhibitors (1% NP-40, 50 mM Tris-HCl pH 7.4, 0.1% SDS, 150 mM NaCl, 0.5% sodium deoxycholate, 1 mM EDTA pH 8, 10 mM sodium fluoride, 1 mM sodium orthovanadate and protease inhibitor cocktail). Proteins were then quantified from cleared lysates using DC Protein Assay (Bio-Rad), and equal amounts of each sample were analyzed by western blotting. The top half portion of the blot was used for Coomassie Brilliant Blue R-250 staining to assess total protein loading and the bottom half was used for immunoblotting with the anti-Flag antibody to assess ARL14^3×Flag^ expression.

### PLA

Cells were fixed and permeabilized as described for immunofluorescence staining. PLA was performed using the Duolink *In Situ* Detection Reagents Red (DUO92008, Sigma-Aldrich) using the anti-mouse MINUS (DUO92004, Sigma-Aldrich) and the anti-rabbit PLUS (DUO92002, Sigma-Aldrich) probes. Images were acquired with a DM6 upright microscope (Leica Microsystems, Ontario, Canada) equipped with an ORCAflash 4.0 V.2 monochromatic camera (Hamamatsu Photonics, Bridgewater, NJ, USA). PLA dots were counted using FIJI (Schindelin et al., 2012) (v2.0.0-rc-69/1.52k).

### Measurement of PLD activity using IMPACT

For IMPACT, cells were plated onto a 24-well plate and protein expression was induced overnight with 1 µg/ml of tetracycline. Cells were pre-treated with FIPI (750 nM, 5282455MG, Millipore Sigma) or DMSO control for 30 min, followed by 3-azidopropanol (1 mM, synthesized in-house as in Yuan et al., 2012) and phorbol 12-myristate 13-acetate (100 nM, P8139, Millipore Sigma) for 30 min. Cells were washed three times with PBS and incubated with BCN-BODIPY (1 µM, synthesized as in Alamudi et al., 2016) diluted in Tyrode's HEPES buffer (135 mM NaCl, 5 mM KCl, 1.8 mM CaCl_2_, 1 mM MgCl_2_, 1 mg/ml glucose, 1 mg/ml BSA, 20 mM HEPES, pH 7.4) for 30 min at 37°C. Cells were rinsed twice with PBS and split into three wells of a 96-well U-bottom plate. The plate was centrifuged at 1000 ***g*** for 2 min and cells were rinsed twice with PBS. Cells were fixed with 4% paraformaldehyde in PBS followed by two additional PBS washes. Cells were resuspended in fluorescence-activated cell sorting (FACS) buffer (0.5% FBS in PBS) for FACS analysis, which was performed on a Attune NxT analyzer (Thermo Fisher Scientific).

### Statistical analyses

All quantification graphs were derived from Prism (GraphPad Software, v7.0a). Statistical significance was determined using two-tailed unpaired Student’s *t*-tests or one-way ANOVA, followed by Bonferroni's multiple comparison test, where *, ** and *** represent *P*<0.05, *P*<0.001 and *P*<0.0001, respectively. Sample sizes and number of replicates are mentioned in the figure legends.

### Raw data

Source files used for western blots and agarose gels throughout the study are provided in Fig. S8. The raw data for the MS experiments are presented in Table S6 and for the BioIDs in Table S7 and S8.

## Supplementary Material

10.1242/joces.262140_sup1 Supplementary information

Table S6. AP-MS results

Table S7 BioID on WT ARFs/ARLs

Table S8. BioID on active ARFs/ARLs
