# Supplementary material for: Mapping the global interactome of the ARF family reveals spatial organization in cellular signaling pathways
Source: J Cell Sci. 2024 May 14;137(9):jcs262140. doi: 10.1242/jcs.262140 (PMC11166204; doi:10.1242/jcs.262140)
Supplement: Supplementary information [file joces-137-262140-s1.pdf]

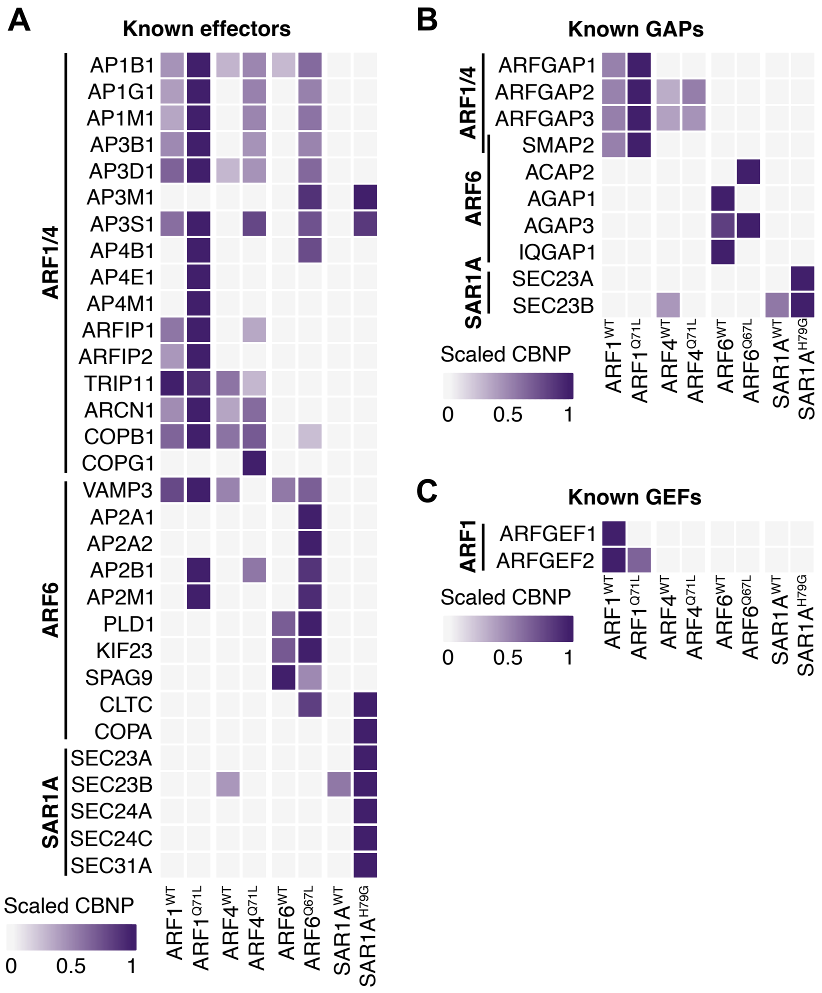

Fig. S1. The activated ARF family members allow for effectors and GAPs enrichment. Heatmaps representing the known **(A)** effectors, **(B)** GAPs and **(C)** GEFs identified in BioID performed in FlpIn T-REx HeLa cells expressing the indicated baits. The scale shows the normalized spectral counts (CBNP) where the darker color is indicative of a higher number of spectral counts.

Journal of Cell Science • Supplementary information

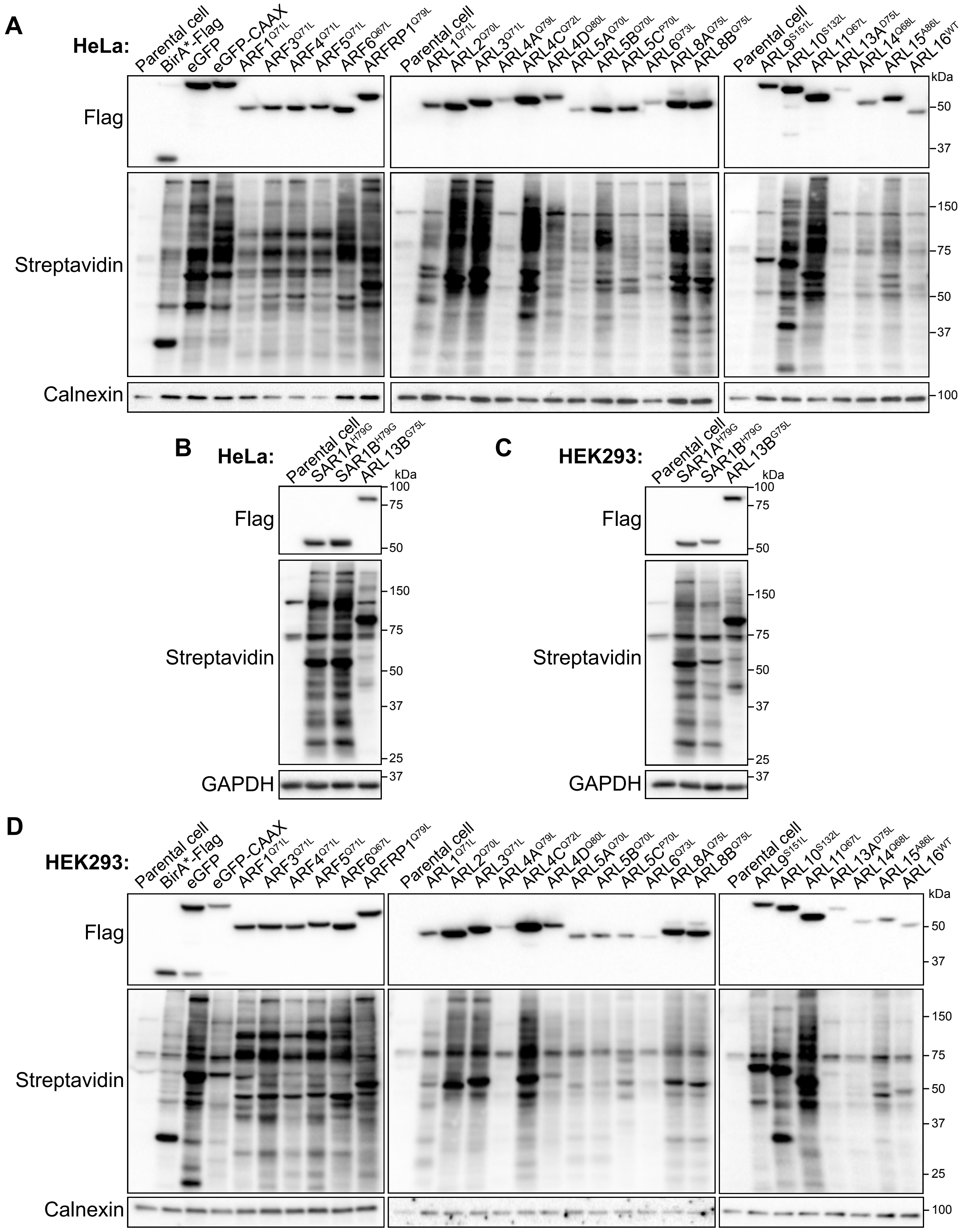

Fig. S2. Validation of the expression and biotinylation of ARF-BirA*-Flag cell lines. (A) and **(B)** Western blot of FlpIn T-REx HeLa cells expressing the indicated constitutively active ARF-BirA*-Flag construct treated for 24 hours with tetracycline and biotin. Loading controls are calnexin in (A) and GAPDH in (B). Data are representative of n=2 experiments. **(C)** and **(D)** Western blot of FlpIn T-REx HEK293 cells expressing the indicated constitutively active ARF-BirA*-Flag construct treated for 24 hours with tetracycline and biotin. Loading controls are GAPDH in (C) and calnexin in (D). Data are representative of n=2 experiments.

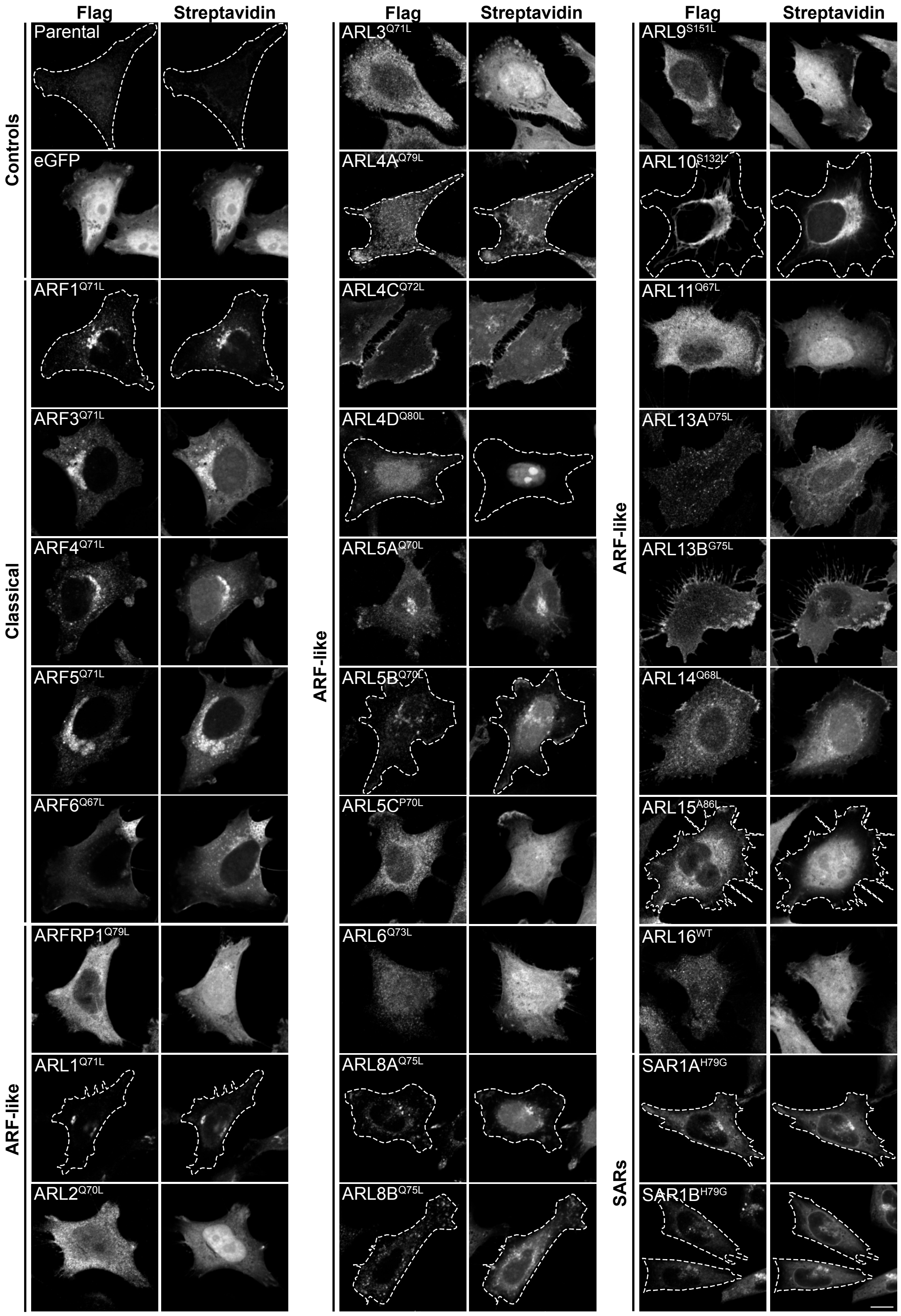

Fig. S3. Validation of the expression and biotinylation of ARF-BirA*-Flag HeLa cells. Confocal microscopy images of immunostaining against Flag (Alexa Fluor 488) and streptavidin (Alexa Fluor 647-Streptavidin) in FlpIn T-REx HeLa cells expressing the indicated constitutively active ARF-BirA*-Flag construct or control treated for 24 hours with tetracycline and biotin. Parental cells were used as control. When necessary, F-actin staining (Alexa Fluor 568-Phalloidin) (not shown) was used to delineate the cell outline. Images are representative of n=3 independent experiments. Bar, 10 µm.

Journal of Cell Science • Supplementary information

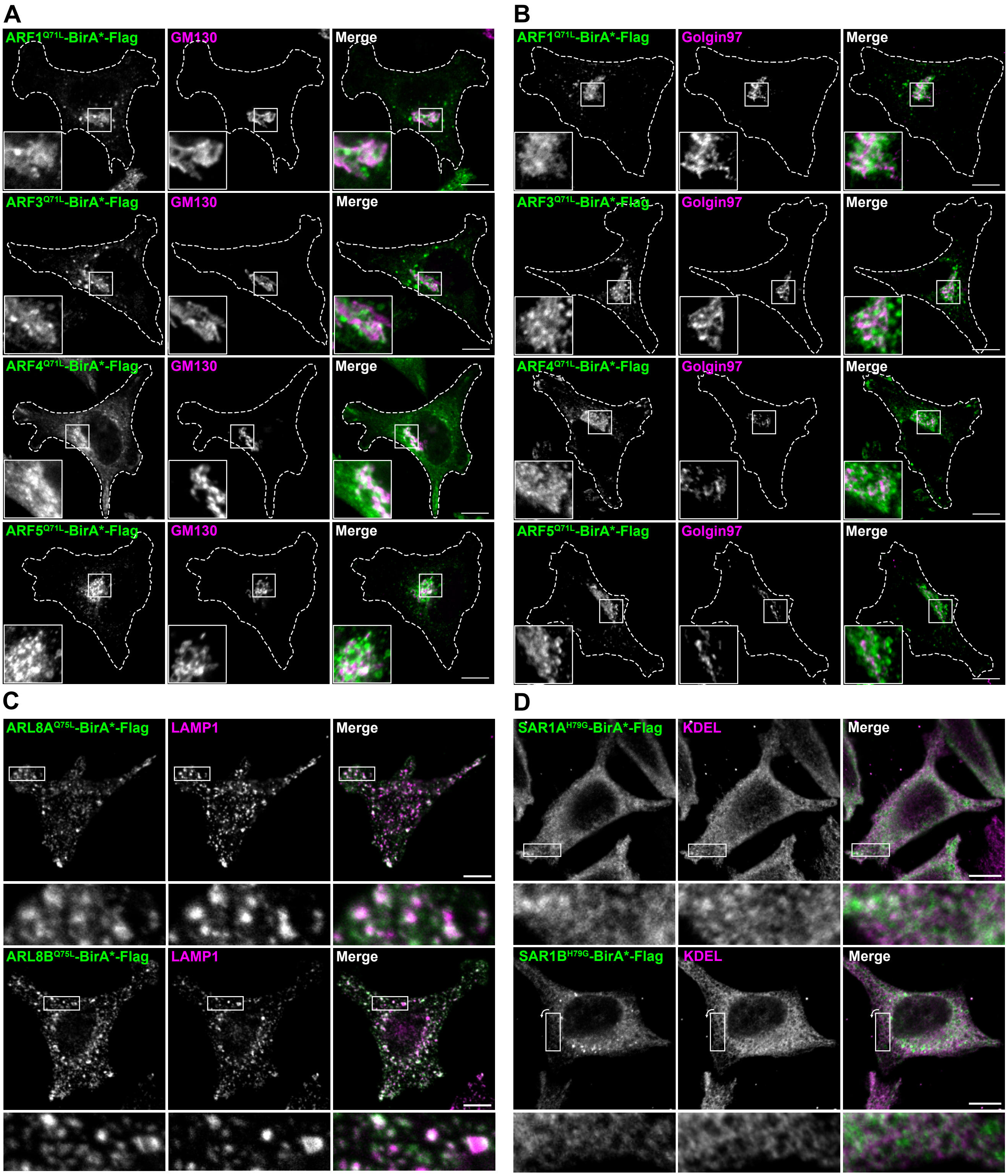

Fig. S4. Validation of the localization of well-established ARF family members. Confocal images of co-immunostaining using anti-Flag and **(A)** anti-GM130 (*cis*-Golgi marker), **(B)** anti-Golgin97 (*trans*-Golgi marker), **(C)** anti-LAMP1 (lysosomes marker) or **(D)** anti-KDEL (the ER lumen marker). FlpIn T-REx HeLa cells were fixed and stained 24 hours after induction expression of the indicated ARF-BirA*-Flag constructs with tetracycline. Insets represent ~2.5x magnification (A-B) and ~4.4 magnification (C-D). Images are representative of n=3 independent experiments. Bars, 10 µm.

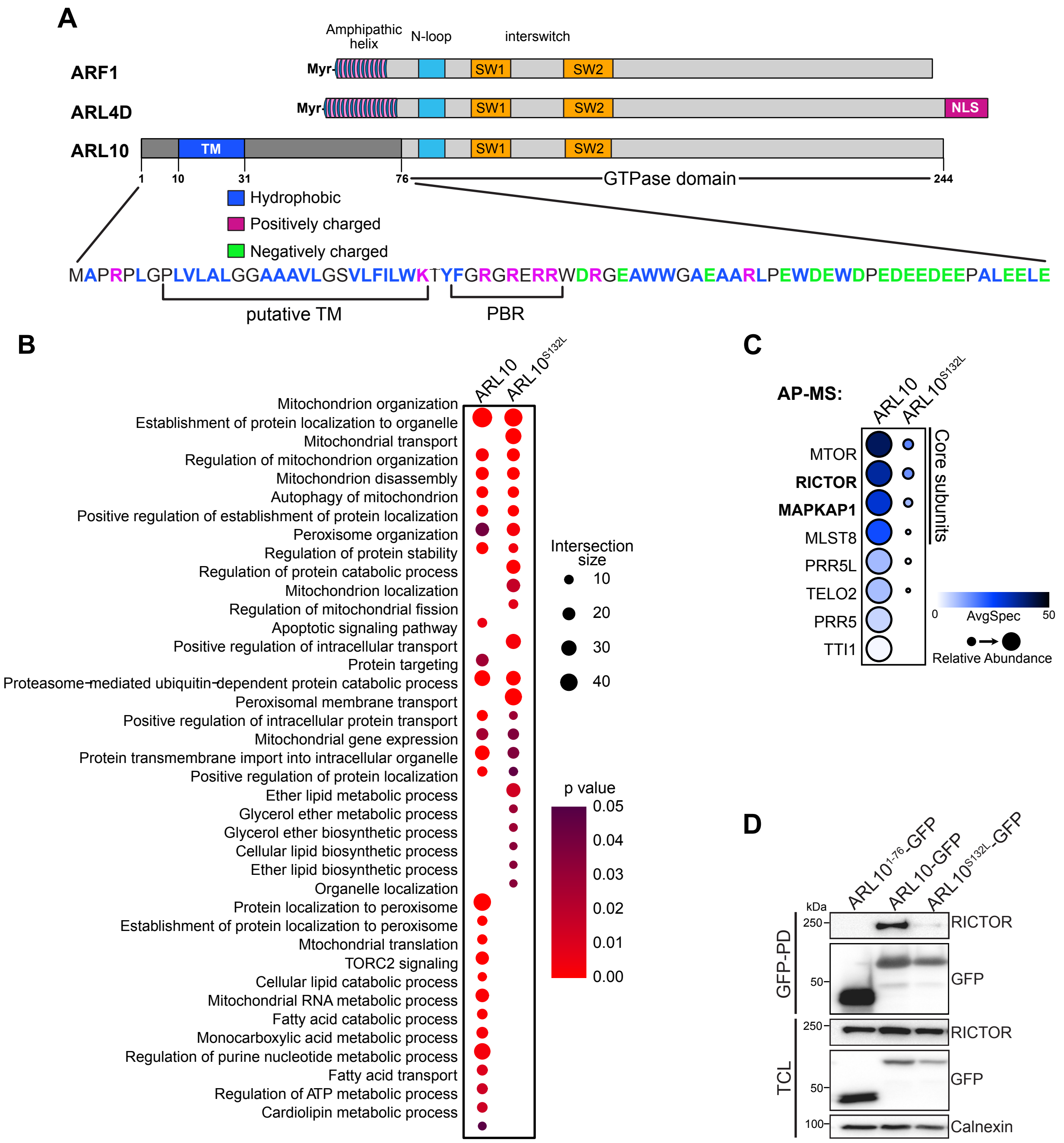

Journal of Cell Science • Supplementary information

Fig. S5. ARL10 is involved in TORC2 signaling and interacts with RICTOR. (A) Schematic representation of ARL4D and ARL10 GTPase features as compared to ARF1. Both ARL4D and ARL10 contain the core ARF GTPase region (in light grey) that includes the N-loop, switch 1 and switch 2 regions. ARL4D contains a nuclear localization signal (NLS). The Myristoylated (Myr) amphipathic helix is conserved in ARL4D but is replaced in ARL10 by a predicted transmembrane (TM) domain enriched in hydrophobic residues followed by a stretch of positively charged (polybasic region, PBR) amino acids region. **(B)** Overrepresentation analyses of GO biological processes enriched from the AP-MS of ARL10-GFP (WT) and constitutively active (S132L) in FlpIn T-REx HeLa cells. The intersection size represents the number of proteins associated with a biological process. **(C)** Dotplot showing AP-MS protein-protein interactions of the mTORC2 complex retrieved in ARL10-GFP (WT) and active (S132L) in FlpIn T-REx HeLa cells. Darker circle color represents higher spectral counts while circle size represents relative abundance. AvgP ≥ 0.95. **(D)** Co-immunoprecipitation of RICTOR by pulldown (PD) of ARL10-GFP WT or constitutively active in FlpIn T-REx HeLa cells. The GFP targeted to mitochondria generated by fusion of ARL101-76 to GFP was used as control. Calnexin was used as loading control in the total cell lysate (TLC). Data are representative of n=3 experiments.

Journal of Cell Science • Supplementary information

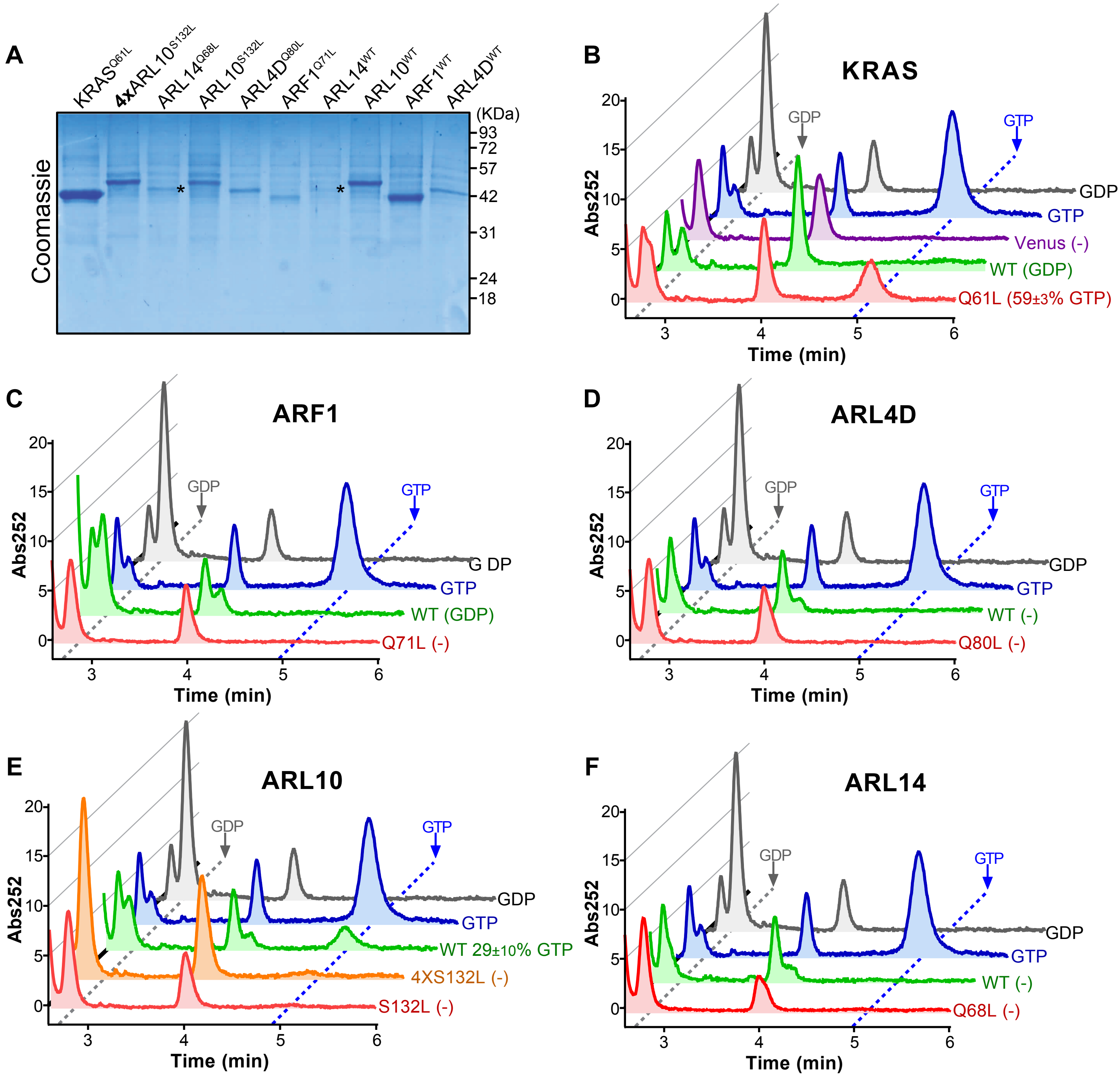

Fig. S6. Constitutively active mutants of ARF and RAS GTPases presents differential nucleotide binding. (A) Coomassie stanning showing the purified fusion proteins used in (B) The asterisk (*) indicates the bands of interest for ARL14. **(B-F)** Ion-pair reversed-phase high-performance liquid chromatography performed on Venus-tagged proteins in their wild-type (in green) or constitutively active form (in red) purified after overexpression in HEK293T cells. Nucleotide alone (GDP in black and GTP in blue) and overexpression of an empty vector (Venus in purple) were used as controls. Absorbance at 252 nm was measured for 6 minutes.

Journal of Cell Science • Supplementary information

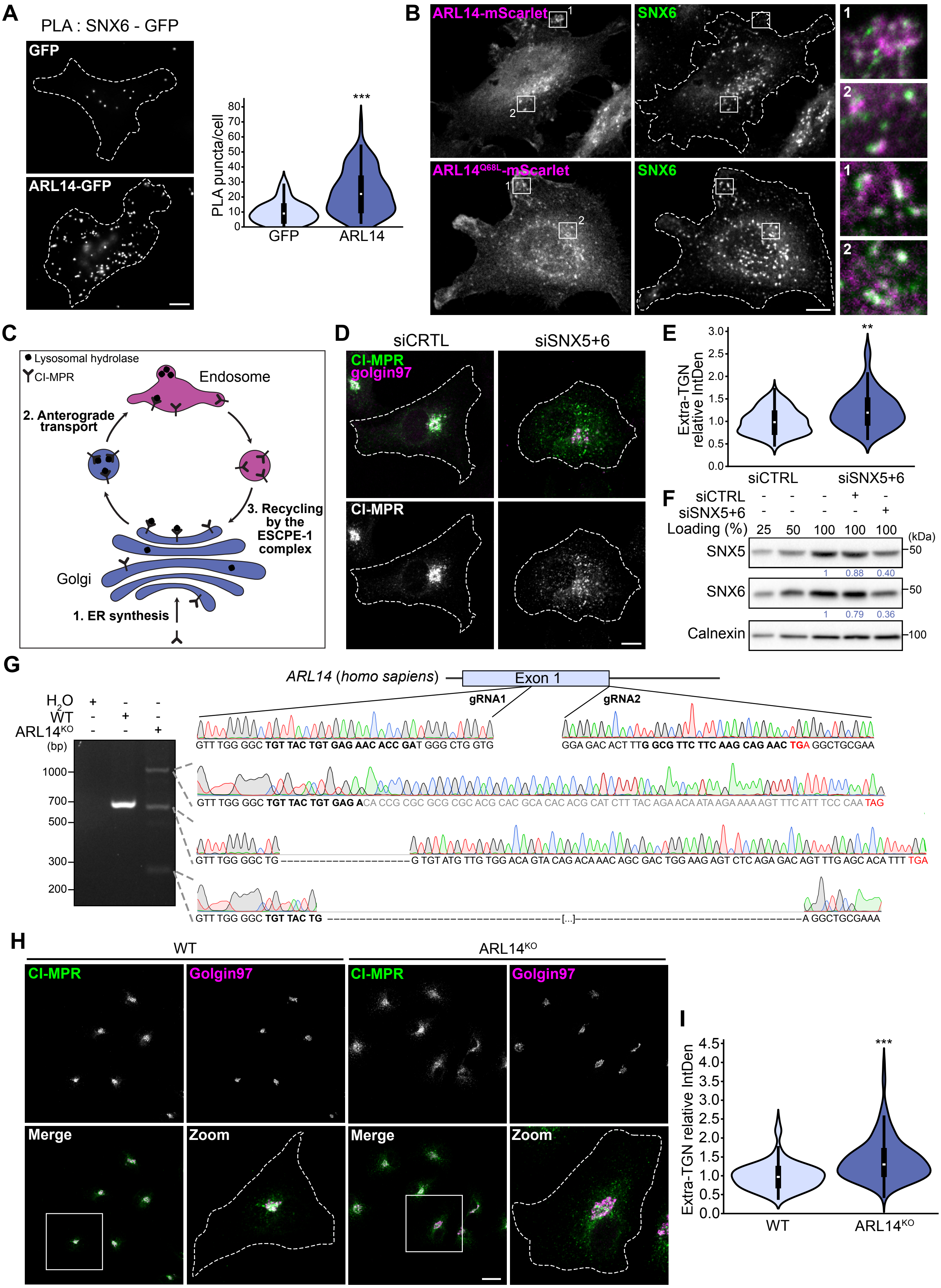

Journal of Cell Science • Supplementary information

Fig. S7. SNX6 is a close proximity interactor of ARL14. (A) PLA of endogenous SNX6 and overexpressed ARL14 WT tagged with GFP performed in FlpIn T-REx HeLa cells. Cells expressing GFP alone were used as a control. Bar, 10 µm. Violin plot showing the quantification of the number of PLA puncta per cell for each condition. Data are representative of n=3 independent experiments (total of 90 cells per condition). *P* value was calculated by Student *t*-test; ****P*< 0.0001. **(B)** SNX6 immunostaining of FlpIn T-REx HeLa expressing ARL14-mScarlet WT or constitutively active (Q68L). Merged images represents a ~4.2x magnification. Images are representative of n=3 independent experiments. Bar, 10 µm. **(C)** Schematic representation of CI-MPR trafficking. **(D)** Co-immunostainings using anti-Golgin97 as TGN marker and anti-CI-MPR of HeLa cells transfected with siCTRL or siSNX5 and siSNX6. Bar, 10µm. **(E)** Violin plot showing the quantification of the integrated density (IntDen) outside the TGN relative to the control for each condition. Data are representative of n=3 independent experiments (total of 90 cells per condition). *P* value was calculated by Student *t*-test; ***P*< 0.001. **(F)** Western blot showing SNX5 and SNX6 knock-down after 72h of treatment with the indicated siRNAs. Calnexin was used as loading control. Data are representative of n=3 independent experiments and quantification are shown under SNX blots. **(G)** Sequencing of the *ARL14* knock-out clone in HeLa cells. Sequences for the two gRNAs used are represented in bold and the STOP codons are identified in red. Hela cells have three copies of this region as shown by the three bands in the agarose gel. The highest band shows an insertion of a small sequence that led to a STOP codon addition. The middle band represents the generation of a single cut which shifted the reading frame and led to a STOP codon addition. The lowest band represents two cuts that led to the deletion of the region between gRNA 1 and 2. **(H)** Co-immunostainings using anti-Golgin97 as TGN marker and anti-CI-MPR in HeLa WT and ARL14 knock-out cells. Dotted lines represent cell outline delimited using F-actin staining (not shown). Zoomed in images represents a ~2.8x magnification. Bar, 10µm. **(I)** Violin plot showing the quantification of the integrated density (IntDen) outside the TGN relative to the control for each condition presented in (H). Data are representative of n=3 independent experiments (total of 120 cells per condition). *P* value was calculated by Student *t*-test; ****P*< 0.0001.

Journal of Cell Science • Supplementary information

Table S1. List of baits used for BioID experiments

	Bait	Bait Mutation	NCBI Reference Sequence or Sequence
Classical	ARF1	Q71L	NM_001024227
	ARF1	WT	NM_001024227
	ARF3	Q71L	NM_001659
	ARF4	Q71L	NM_001660
	ARF4	WT	NM_001660
	ARF5	Q71L	NM_001662
	ARF6	Q67L	NM_001663
	ARF6	WT	NM_001663
ARF-Like	ARL1	Q71L	NM_001177
	ARFRP1	Q79L	NM_003224
	ARL2	Q70L	NM_001667
	ARL3	Q71L	NM_004311
	ARL4A	Q79L	NM_005738
	ARL4C	Q72L	NM_005737
	ARL4D	Q80L	NM_001661
	ARL5A	Q70L	NM_012097
	ARL5B	Q70L	NM_178815
	ARL5C	P70L	NM_001143968
	ARL6	Q73L	NM_001323513.2
	ARL8A	Q75L	NM_138795
	ARL8B	Q75L	NM_018184
	ARL9	S151L	NM_001363794.2
	ARL10	S132L	NM_173664
	ARL11	Q67L	NM_138450
	ARL13A	D75L	NM_001162490
	ARL13B	G75L	NM_182896
	ARL14	Q68L	NM_025047
	ARL15	A86L	NM_019087
	ARL16	WT	NM_001040025.3
SAR	SAR1A	H79G	NM_001142648
	SAR1A	WT	NM_001142648
	SAR1B	H79G	NM_001033503
Control	Empty vector	NA	NA
	EGFP	NA	ATGGTGAGCAAGGGCGAGGAGCTGTTCACCGGGGTGGTGCCCATCCTGGTC<br>GAGCTGGACGGCGACGTAAACGGCCACAAGTTCAGCGTGTCCGGCGAGGG<br>CGAGGGCGATGCCACCTACGGCAAGCTGACCCTGAAGTTCATCTGCACCAC<br>CGGCAAGCTGCCCGTGCCCTGGCCCACCCTCGTGACCACCCTGACCTACG<br>GCGTGCAGTGCTTCAGCCGCTACCCCGACCACATGAAGCAGCACGACTTCT<br>TCAAGTCCGCCATGCCCGAAGGCTACGTCCAGGAGCGCACCATCTTCTTCA<br>AGGACGACGGCAACTACAAGACCCGCGCCGAGGTGAAGTTCGAGGGCGAC<br>ACCCTGGTGAACCGCATCGAGCTGAAGGGCATCGACTTCAAGGAGGACGGC<br>AACATCCTGGGGCACAAGCTGGAGTACAACTACAACAGCCACAACGTCTAT<br>ATCATGGCCGACAAGCAGAAGAACGGCATCAAGGTGAACTTCAAGATCCGC<br>CACAACATCGAGGACGGCAGCGTGCAGCTCGCCGACCACTACCAGCAGAA<br>CACCCCCATCGGCGACGGCCCCGTGCTGCTGCCCGACAACCACTACCTGA<br>GCACCCAGTCCGCCCTGAGCAAAGACCCCAACGAGAAGCGCGATCACATG<br>GTCCTGCTGGAGTTCGTGACCGCCGCCGGGATCACTCTCGGCATGGACGAG<br>CTGTACAAGTAA
	EGFP-CAAX	NA	ATGGTGAGCAAGGGCGAGGAGCTGTTCACCGGGGTGGTGCCCATCCTGGTC<br>GAGCTGGACGGCGACGTAAACGGCCACAAGTTCAGCGTGTCCGGCGAGGG<br>CGAGGGCGATGCCACCTACGGCAAGCTGACCCTGAAGTTCATCTGCACCAC<br>CGGCAAGCTGCCCGTGCCCTGGCCCACCCTCGTGACCACCCTGACCTACG<br>GCGTGCAGTGCTTCAGCCGCTACCCCGACCACATGAAGCAGCACGACTTCT<br>TCAAGTCCGCCATGCCCGAAGGCTACGTCCAGGAGCGCACCATCTTCTTCA<br>AGGACGACGGCAACTACAAGACCCGCGCCGAGGTGAAGTTCGAGGGCGAC<br>ACCCTGGTGAACCGCATCGAGCTGAAGGGCATCGACTTCAAGGAGGACGGC<br>AACATCCTGGGGCACAAGCTGGAGTACAACTACAACAGCCACAACGTCTAT<br>ATCATGGCCGACAAGCAGAAGAACGGCATCAAGGTGAACTTCAAGATCCGC<br>CACAACATCGAGGACGGCAGCGTGCAGCTCGCCGACCACTACCAGCAGAA<br>CACCCCCATCGGCGACGGCCCCGTGCTGCTGCCCGACAACCACTACCTGA<br>GCACCCAGTCCGCCCTGAGCAAAGACCCCAACGAGAAGCGCGATCACATG<br>GTCCTGCTGGAGTTCGTGACCGCCGCCGGGATCACTCTCGGCATGGACGAG<br>CTGTACAAGAAGAAGAAAAAGAAGTCAAAGACAAAGTGTGTAATTATGTAG

Table S2. List of antibodies used in this study

Primary Antibodies for WB	Source	Cat#	Dilution	Incubation	
Mouse anti-calnexin (E10)	Santa Cruz Biotechnology	sc-46669	1:1000	overnight 4°C	
anti-Flag-HRP	Sigma-Aldrich	A8592	1:8000	1h room temp	
rabbit anti-GAPDH	Santa Cruz Biotechnology	SC-25778	1:5000	1h room temp	
Rabbit anti-GFP	Thermo Fisher Scientific	A-11122	1:2000	overnight 4°C	
Rabbit anti-Rictor (D16H9)	Cell Signaling Technology	9476S	1:1000	overnight 4°C	
Rabbit anti-SNX5	AbCam	ab180520	1:500	overnight 4°C	
Mouse anti-SNX6	Santa Cruz Biotechnology	sc-365965	1:1000	overnight 4°C	
Rabbit anti-M6PR	AbCam	ab124767	1:10 000	overnight 4°C	
Rabbit anti-Golgin 97	Proteintech	12640-1-AP	1:2000	overnight 4°C	
Rabbit anti-KDEL	AbCam	ab12223	1:800	1h room temp	
anti-Streptavidin-HRP	BD Biosciences	554066	1:25000	1h room temp	
**Primary Antibodies for IF and PLA**	**Source**	**Cat#**	**Dilution**	**Incubation**	**Application**
Rabbit anti-DYKDDDDK Tag (D6W5B)	Cell Signaling Technology	14793S	1:1000	1h 37°C	IF
Mouse anti-Golgin 97 (CDFX)	Santa Cruz Biotechnology	sc-59820	1:000	1h 37°C	IF
Rabbit anti-PLD1	Cell Signaling Technology	3832S	1:100	1h 37°C	IF
Mouse anti-SNX1	BD Biosciences	611482	1:100	1h 37°C	IF
Mouse anti-SNX6	Santa Cruz Biotechnology	sc-365965	1:100	1h 37°C	IF
Mouse anti-TOM20	Proteintech	66777-1-Ig	1:500	1h 37°C	IF
Rabbit anti-PEX14	Sigma-Aldrich	HPA049231	1:150	1h 37°C	IF
Rabbit anti-GM130	Proteintech	11308-1-AP	1:200	1h 37°C	IF
Rabbit anti-M6PR	AbCam	ab124767	1:1000	1h 37°C	IF
Mouse anti-HA-tag	Cell Signaling Technology	2367S	1:500	1h 37°C	PLA
Rabbit anti-GFP	Thermo Fisher Scientific	A-11122	84:20:00	overnight 4°C	PLA
**Secondary Antibodies**	**Source**	**Cat#**	**Dilution**	**Incubation**	**Application**
Secondary IgG-HRP anti-mouse	Sigma-Aldrich	A4416	1:5000	1h room temp	WB
Secondary IgG-HRP anti-rabbit	Santa Cruz	SC-2357	1:5000	1h room temp	WB
Alexa Fluor 488 chicken anti-rabbit IgG	Thermo Fisher Scientific	A21441	1:500	30min room temp	IF
Alexa Fluor 488 chicken anti-mouse IgG	Thermo Fisher Scientific	A21200	1:500	30min room temp	IF
Alexa Fluor 568 goat anti-rabbit IgG	Thermo Fisher Scientific	A11011	1:500	30min room temp	IF
Alexa Fluor 568 goat anti-mouse IgG	Thermo Fisher Scientific	A11031	1:500	30min room temp	IF
Alexa Fluor 633 goat anti-mouse IgG	Thermo Fisher Scientific	A21050	1:500	30min room temp	IF
**Staining reagents**	**Source**	**Cat#**	**Dilution**	**Incubation**	
Alexa Fluor 568 Phalloidin	Thermo Fisher	A22284	1:1000	1h room temp	
Alexa Fluor 647 Streptavidin	Thermo Fisher	S32357	1:1000	1h room temp	

Journal of Cell Science • Supplementary information

Table S3. List of plasmids used in this study

Plasmid	Source/Ref.	Application
pcDNA5-pDEST-FRT-BirA*-Flag-CT	A.C.Gingras, Lunenfeld Tanenbaum Research Institute (Couzens et al., 2013)	BioID, WB
pcDNA5-pDEST-FRT-ARF1Q71L-BirA*-Flag	J.F. Coté, IRCM (Patel et al., 2011)	BioID, IF, WB
pcDNA5-pDEST-FRT-ARF3^{Q71L}-BirA*-Flag	J.F. Coté, IRCM (Patel et al., 2011)	BioID, IF, WB
pcDNA5-pDEST-FRT-ARF4^{Q71L}-BirA*-Flag	J.F. Coté, IRCM (Patel et al., 2011)	BioID, IF, WB
pcDNA5-pDEST-FRT-ARF5^{Q71L}-BirA*-Flag	J.F. Coté, IRCM (Patel et al., 2011)	BioID, IF, WB
pcDNA5-pDEST-FRT-ARF6^{Q67L}-BirA*-Flag	J.F. Coté, IRCM (Patel et al., 2011)	BioID, IF, WB
pcDNA5-pDEST-FRT-ARL1^{Q71L}-BirA*-Flag	J.F. Coté, IRCM (Patel et al., 2011)	BioID, IF, WB
pcDNA5-pDEST-FRT-ARFRP1^{Q79L}-BirA*-Flag	J.F. Coté, IRCM (Patel et al., 2011)	BioID, IF, WB
pcDNA5-pDEST-FRT-ARL2^{Q70L}-BirA*-Flag	J.F. Coté, IRCM (Patel et al., 2011)	BioID, IF, WB
pcDNA5-pDEST-FRT-ARL3^{Q71L}-BirA*-Flag	J.F. Coté, IRCM (Patel et al., 2011)	BioID, IF, WB
pcDNA5-pDEST-FRT-ARL4a^{Q79L}-BirA*-Flag	J.F. Coté, IRCM (Patel et al., 2011)	BioID, IF, WB
pcDNA5-pDEST-FRT-ARL4c^{Q72L}-BirA*-Flag	J.F. Coté, IRCM (Patel et al., 2011)	BioID, IF, WB
pcDNA5-pDEST-FRT-ARL4d^{Q80L}-BirA*-Flag	J.F. Coté, IRCM (Patel et al., 2011)	BioID, IF, WB
pcDNA5-pDEST-FRT-ARL5a^{Q70L}-BirA*-Flag	J.F. Coté, IRCM (Patel et al., 2011)	BioID, IF, WB
pcDNA5-pDEST-FRT-ARL5b^{Q70L}-BirA*-Flag	J.F. Coté, IRCM (Patel et al., 2011)	BioID, IF, WB
pcDNA5-pDEST-FRT-ARL5c^{P70L}-BirA*-Flag	J.F. Coté, IRCM (Patel et al., 2011)	BioID, IF, WB
pcDNA5-pDEST-FRT-ARL6^{Q73L}-BirA*-Flag	This study	BioID, IF, WB
pcDNA5-pDEST-FRT-ARL8a^{Q75L}-BirA*-Flag	J.F. Coté, IRCM (Patel et al., 2011)	BioID, IF, WB
pcDNA5-pDEST-FRT-ARL8b^{Q75L}-BirA*-Flag	J.F. Coté, IRCM (Patel et al., 2011)	BioID, IF, WB
pcDNA5-pDEST-FRT-ARL9^{S151L}-BirA*-Flag	This study	BioID, IF, WB
pcDNA5-pDEST-FRT-ARL10^{S132L}-BirA*-Flag	J.F. Coté, IRCM (Patel et al., 2011)	BioID, IF, WB
pcDNA5-pDEST-FRT-ARL11^{Q67L}-BirA*-Flag	J.F. Coté, IRCM (Patel et al., 2011)	BioID, IF, WB
pcDNA5-pDEST-FRT-ARL13a^{D75L}-BirA*-Flag	J.F. Coté, IRCM (Patel et al., 2011)	BioID, IF, WB
pcDNA5-pDEST-FRT-ARL13b^{G75L}-BirA*-Flag	J.F. Coté, IRCM (Patel et al., 2011)	BioID, IF, WB
pcDNA5-pDEST-FRT-ARL14^{Q68L}-BirA*-Flag	J.F. Coté, IRCM (Patel et al., 2011)	BioID, IF, WB
pcDNA5-pDEST-FRT-ARL15^{A86L}-BirA*-Flag	J.F. Coté, IRCM (Patel et al., 2011)	BioID, IF, WB
pcDNA5-pDEST-FRT-ARL15^{A86L}-BirA*-Flag	J.F. Coté, IRCM (Patel et al., 2011)	BioID, IF, WB
pcDNA5-pDEST-FRT-ARL16-BirA*-Flag	This study	BioID, IF, WB
pcDNA5-pDEST-FRT-SAR1a^{H79G}-BirA*-Flag	J.F. Coté, IRCM (Patel et al., 2011)	BioID, IF, WB
pcDNA5-pDEST-FRT-SAR1b^{H79G}-BirA*-Flag	J.F. Coté, IRCM (Patel et al., 2011)	BioID, IF, WB
pcDNA5-pDEST-FRT-TRIM23^{458I}-BirA*-Flag	J.F. Coté, IRCM (Patel et al., 2011)	BioID, IF, WB
pcDNA5-pDEST-BirA*-Flag-EGFP	J.F. Coté, IRCM (Bagci et al., 2020)	BioID, WB
pcDNA5-pDEST-BirA*-FLAG-EGFP-CAAX	J.F. Coté, IRCM (Bagci et al., 2020)	BioID, WB
pcDNA5-pDEST-FRT-ARL14-EGFP	This study	IF, PLA
pcDNA5-pDEST-FRT-ARL14^{S27N}-EGFP	This study	IF, PLA
pcDNA5-pDEST-FRT-ARL14^{Q68L}-EGFP	This study	IF, PLA
pcDNA5-pDEST-FRT-ARL14-mScarlet	This study	IMPACT
pcDNA5-pDEST-FRT-ARL14^{S27N}-mScarlet	This study	IMPACT
pcDNA5-pDEST-FRT-ARL14^{Q68L}-mScarlet	This study	IMPACT
pcDNA5-pDEST-FRT-ARL10-EGFP	This study	IF, co-IP, AP-MS
pcDNA5-pDEST-FRT-ARL10S91N-EGFP	This study	IF, co-IP, AP-MS
pcDNA5-pDEST-FRT-ARL10^{S132L}-EGFP	This study	IF, co-IP, AP-MS
pCGN-HA-PLD1	M.A. Frohman, Stony Brook Cancer Center (Hammond et al., 1995)	PLA
pOG44	Thermo Fisher Scientific (12536017)	Transfections
pDONR221	Thermo Fisher Scientific (V600520)	Cloning
pcDNA5-pDEST-FRT-mScarlet-CT	This study	Cloning
pcDNA5-pDEST-FRT-EGFP-CT	A.C.Gingras, Lunenfeld Tanenbaum Research Institute (Kean et al., 2011)	Cloning
YFP-hPOP1-C1	This study	Transfections
pGEX-4T-1-ARL14	This study	NMR, ITC
pGEX-4T-1-ARL11	This study	NMR, ITC
pDEST-pcDNA-ARF1-Venus	This study	HPLC
pDEST-pcDNA-ARF1^{Q71L}-Venus	This study	HPLC
pDEST-pcDNA-ARL14-Venus	This study	HPLC
pDEST-pcDNA-ARL14^{Q68L}-Venus	This study	HPLC
pDEST-pcDNA-ARL4D-Venus	This study	HPLC
pDEST-pcDNA-ARL4D^{Q80L}-Venus	This study	HPLC
pDEST-pcDNA-ARL10-Venus	This study	HPLC
pDEST-pcDNA-ARL10^{S132L}-Venus	This study	HPLC
pDEST-pcDNA-KRAS^{Q61L}-Venus	This study	HPLC
pDEST-pcDNA-Venus	This study	HPLC

Journal of Cell Science • Supplementary information

Table S4. List of siRNA used in this study

siRNA	Cat#	Sequence
SNX5	siGENOME Human SNX5 (27131) siRNA - SMART pool M-012524-00-0005	UAACAGAGCUCCUCCGAUA
		GAGCAAAGACGUCAAGUUA
		CAAACAAAGCUCUGGAUAA
		CUACGAAGCCCGACUUUGA
SNX6	siGENOME Human SNX6 (58533) siRNA - SMART pool M-017557-01-0005	GAUGAAGACCUCAAACUUU
		UAAAUCAGCAGAUGGAGUA
		CAAGAAGAGUUGCUGCAUU
		ACUUAGUAGUUAUGCGAGU
siCTRL	siGENOME Non-Targeting siRNA #1 - D-001210-01-20	UAGCGACUAAACACAUCAA

Table S5. List of oligos used in this study

PCR	Target	Application	Forward	Reverse
	mScarlet	KpnI/mScarlet/XhoI PCR amplification	5'-AAGCTTGGTACCATGGTGAGCAAGGGC-3'	5'-CATCATCTCGAGTTACTTGTACAGCTCGTCCATGCC-3'
	ARL10-GFP	Create ARL10 a.a 1-31-GFP	5'-ATGGTGAGCAAGGGCGAGGA-3'	5'-GTAGGTCTTCCAGAGGATGAAGAGCACCG-3'
	ARL10-GFP	Create ARL10 a.a 1-76-GFP	5'-ATGGTGAGCAAGGGCGAGGA-3'	5'-TTCCAGCTCCTCCAGCGCC-3'
	ARL10-GFP	Create ARL10 Δ7-31_GFP	5'-GGCCGCGGCCGAGAG-3'	5'-GAACAGCGGCCGCGG-3'
	ARL10-GFP	Create ARL10 Δ32-76_GFP	5'-CGCGAGGTGCTGGTGC-3'	5'-CTGGTAGGTCTTCCAGAGGATGAAGAGC-3'
	ARL10-GFP	Create ARL10 Δ7-76_GFP	5'-CGCGAGGTGCTGGTGC-3'	5'-CTGCAGCGGCCGCGG-3'
	mARL14	Genotyping ARL14 embryo	5'-GCTGTGCGGTCACTGGAGAA-3'	5'-CCAAAAGTTTGCCGACTCAGC-3'
	mARL14	Genotyping ARL14 embryo in FLAG	5'-GCTGTGCGGTCACTGGAGAA-3'	5'- GTCATGATCTTTATAATCACCGTCATGGTC-3'
	hARL14	Validation of ARL14 KO cells	5'-TCACAGTCTGGGATGTTGGA-3'	5'-AGTGATCATTGCCACAGTTTGC-3'

CRISPR	Name	Application	Sequence
	gRNA_mARL14	To target CRISPR machinery to ARL14	5'-AGTTTCTGAAAAGCTACCGA-3'
	ssDNA_ARL14_3xFlag	Template to insert 3xFlag	5'AATAAAACAGATTTCCCATTTCCACTTCATC ATTGTCTCACTTGTCATCGTCATCCTTGTAGT CGATGTCATGATCTTTATAATCACCGTCATGG TCTTTGTAGTCCCCTCCACTGCCACCTTTCT GCTTGAAGATTGCTAAGGTCTCTCTTGTTCTT
	gRNA1_hARL14_F	To target CRISPR machinery to ARL14	5'-CACCGTGTTACTGTGAGAACACCGA-3'
	gRNA1_hARL14_R	To target CRISPR machinery to ARL14	5'-AAACTCGGTGTTCTCACAGTAACAC-3'
	gRNA2_hARL14_F	To target CRISPR machinery to ARL14	5'-CACCGGCGTTCTTCAAGCAGAACTG-3'
	gRNA2_hARL14_R	To target CRISPR machinery to ARL14	5'-AAACCAGTTCTGCTTGAAGAACGCC-3'

Table S6. AP-MS results

Available for download at

https://journals.biologists.com/jcs/article-lookup/doi/10.1242/jcs.262140#supplementary-data

Table S7 BioID on WT ARFs/ARLs

Available for download at

https://journals.biologists.com/jcs/article-lookup/doi/10.1242/jcs.262140#supplementary-data

Table S8. BioID on active ARFs/ARLs

Available for download at

https://journals.biologists.com/jcs/article-lookup/doi/10.1242/jcs.262140#supplementary-data

Journal of Cell Science • Supplementary information

Figure 5

B)

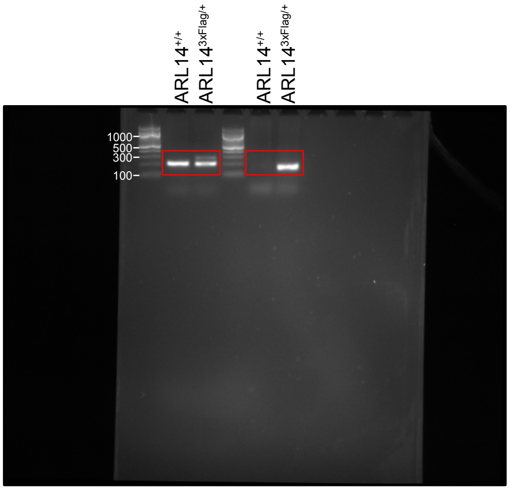

C)

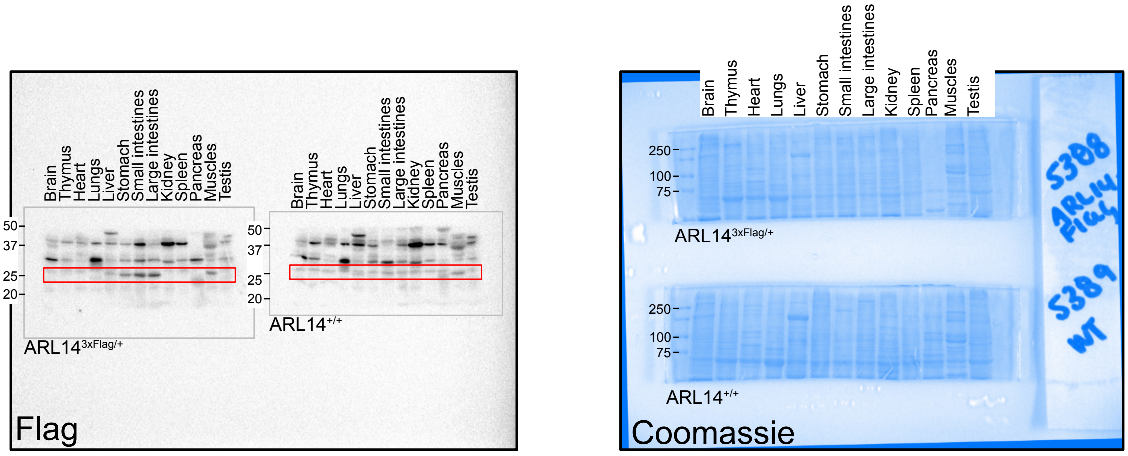

Journal of Cell Science • Supplementary information

Figure 7 D)

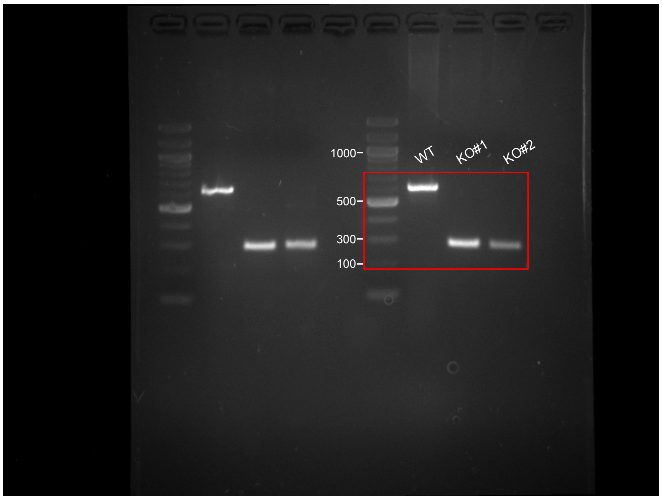
1000
500
300
100
WT
KO#1
KO#2

E)

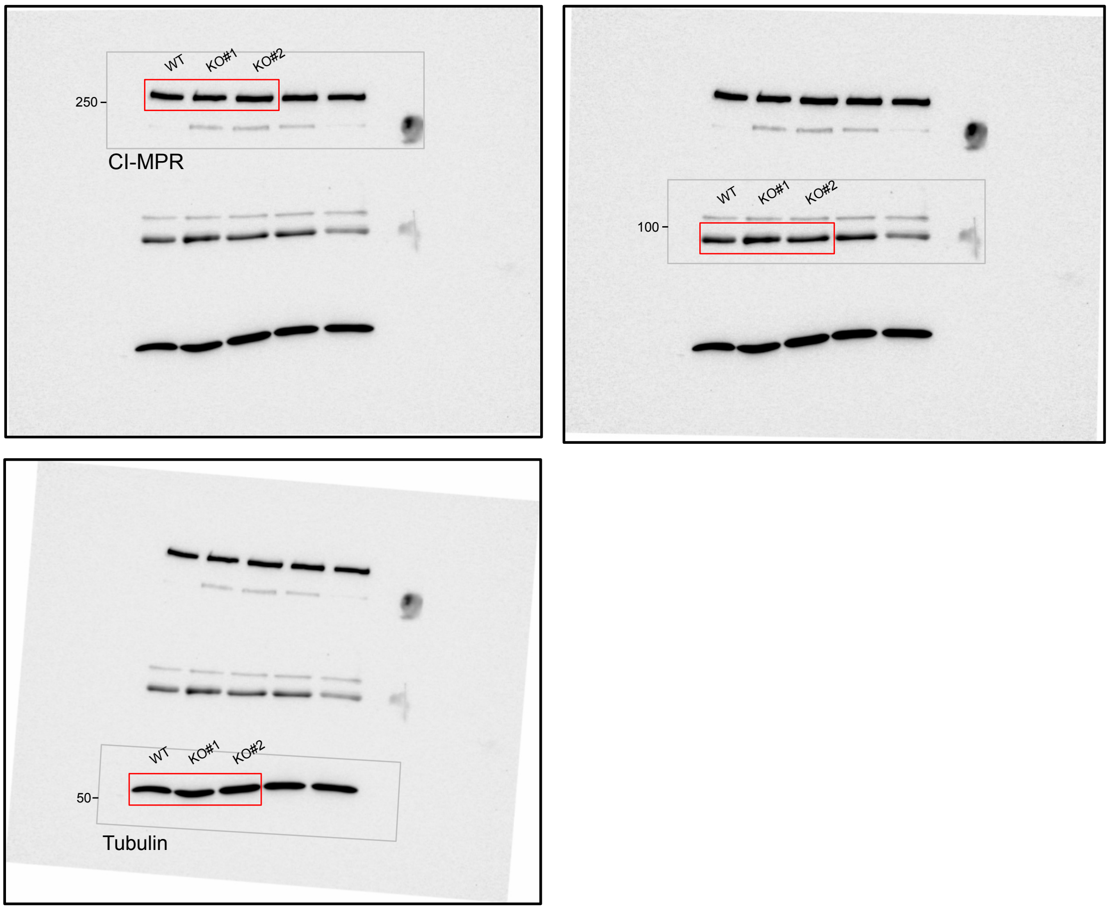
WT
KO#1
KO#2
250
CI-MPR
WT
KO#1
KO#2
100
WT
KO#1
KO#2
50
Tubulin

Figure S2

A)

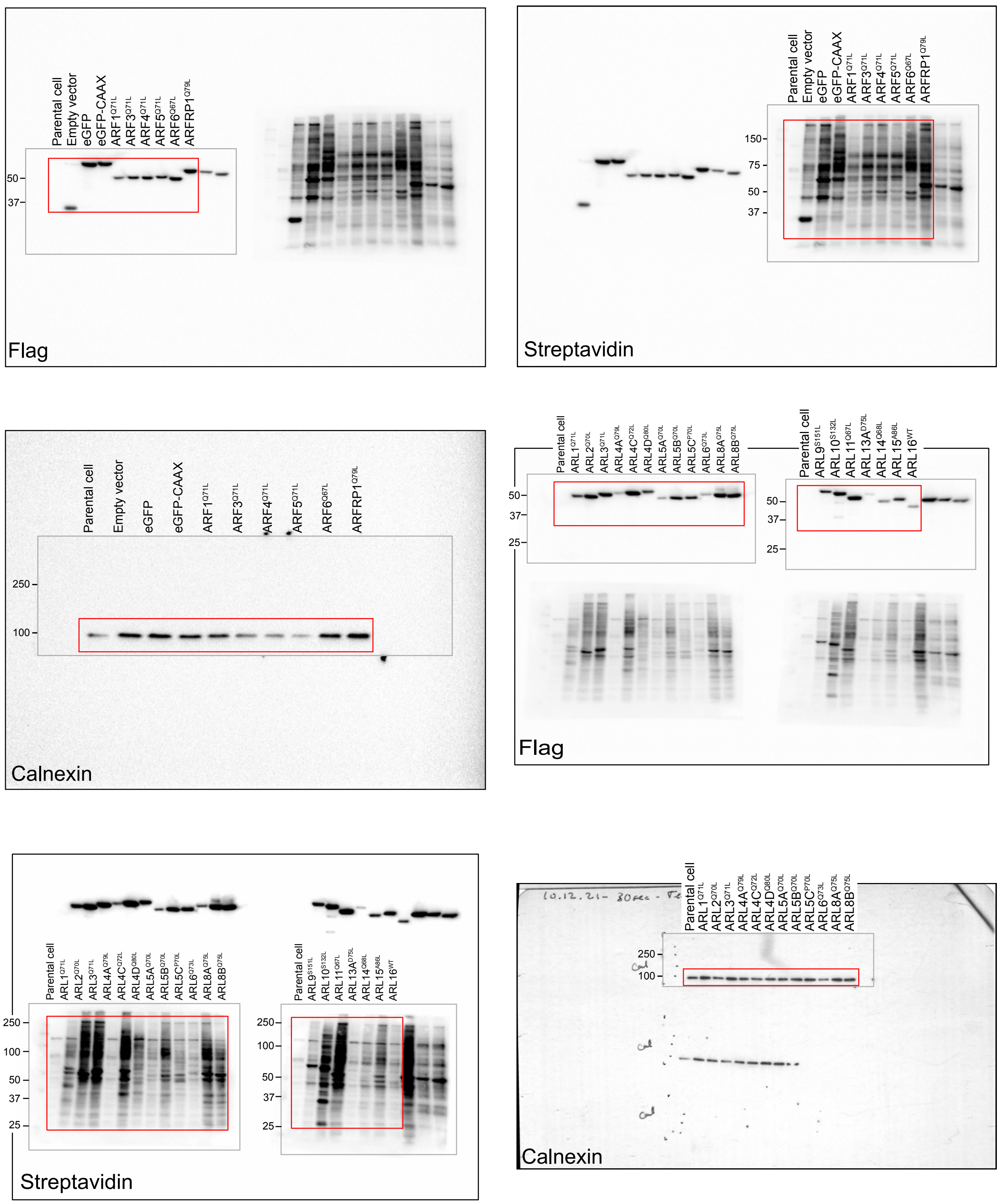

Journal of Cell Science • Supplementary information

Figure S2 B)

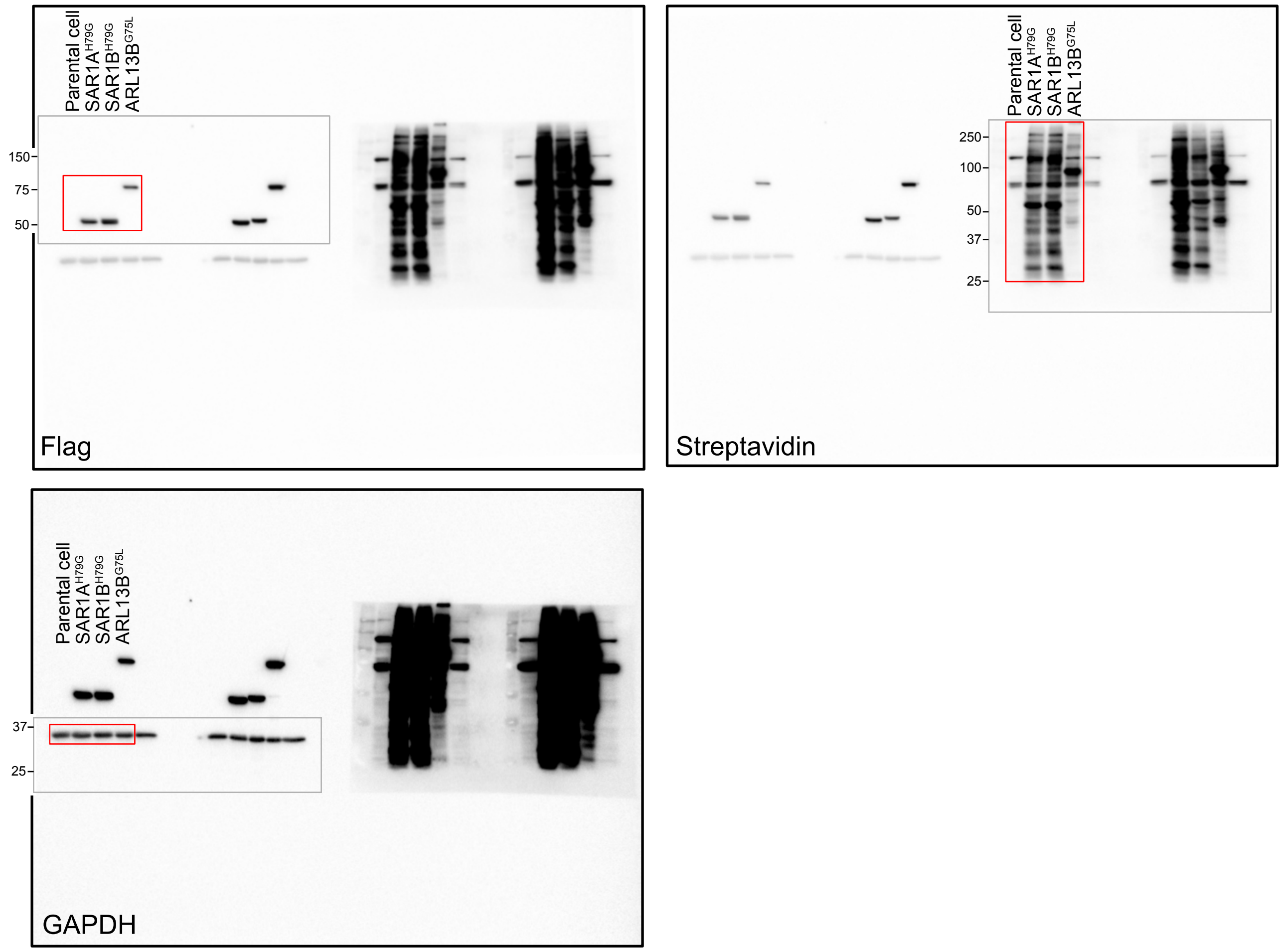

Journal of Cell Science • Supplementary information

Figure S2 C)

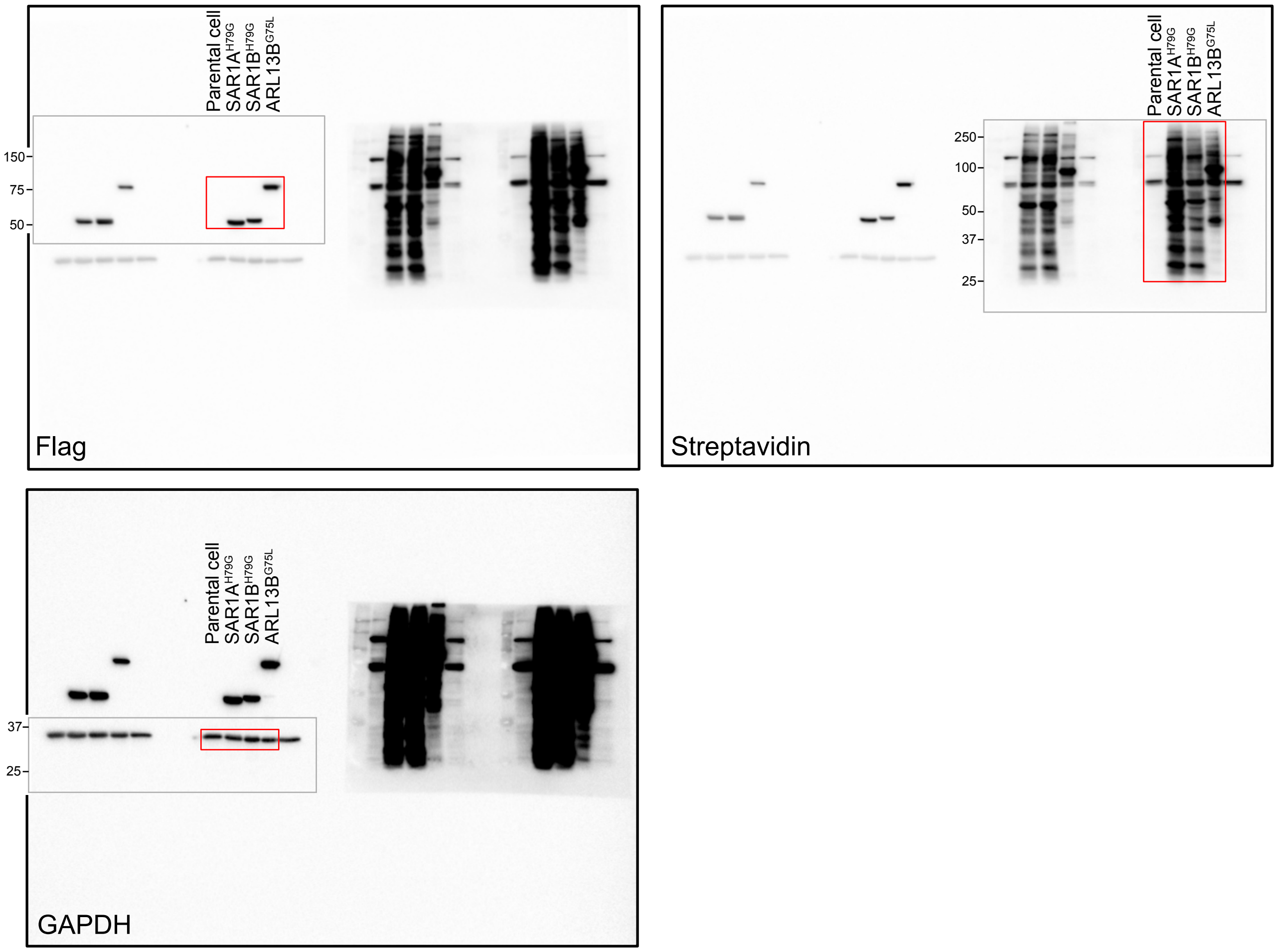

Journal of Cell Science • Supplementary information

Figure S2 D)

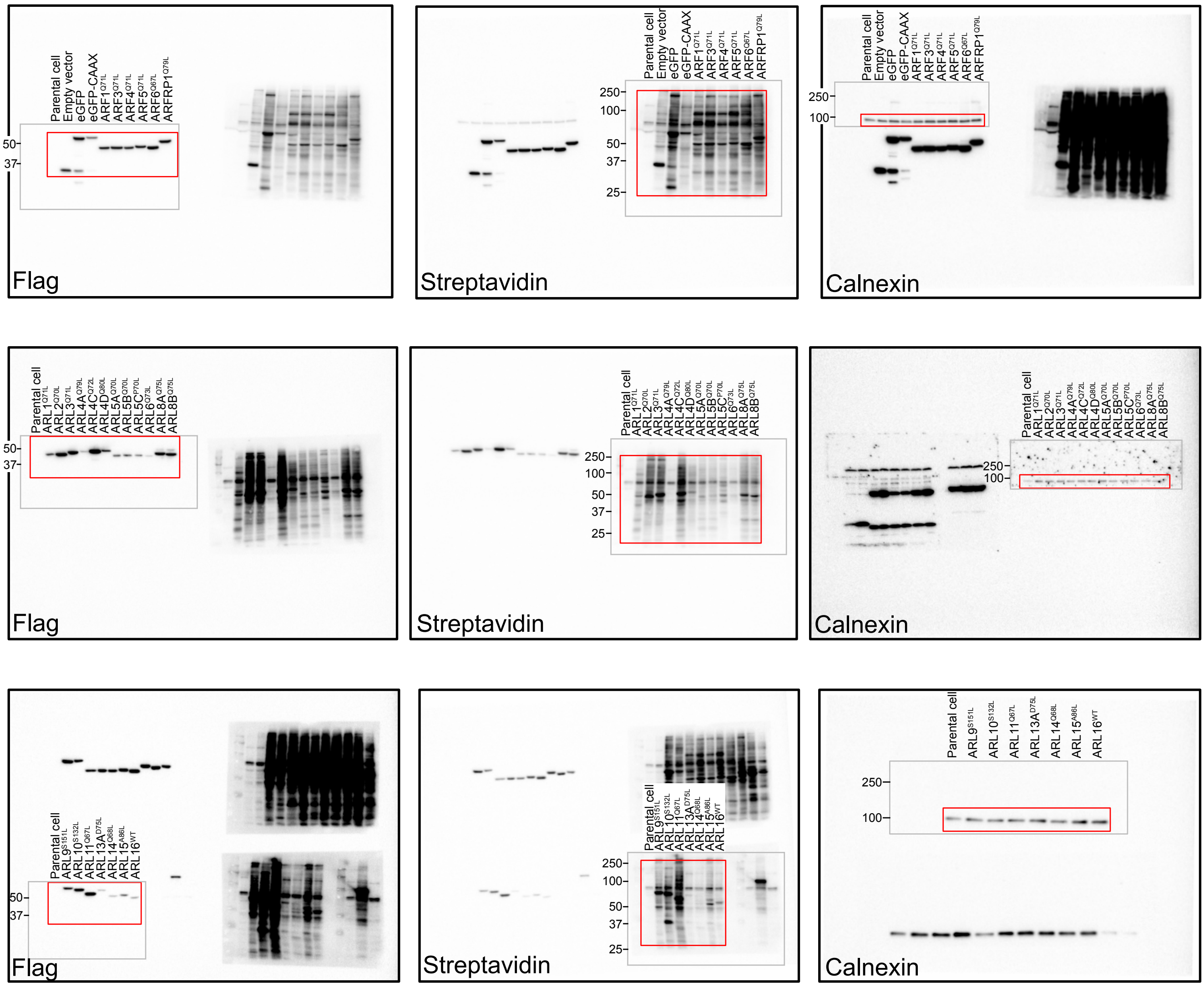

Journal of Cell Science • Supplementary information

Figure S5

lane 1 : Mito-GFP

lane 2: ARL10-GFP

lane 3: ARL10^{S132L}-GFP

lane 4: ARL10^{S91N}-GFP

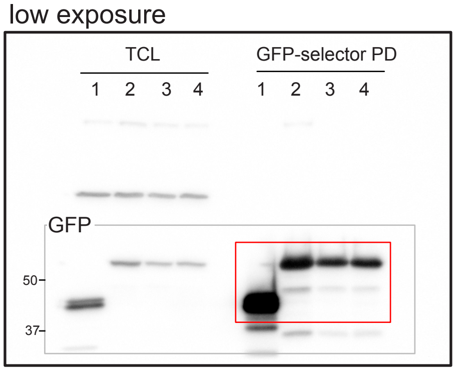

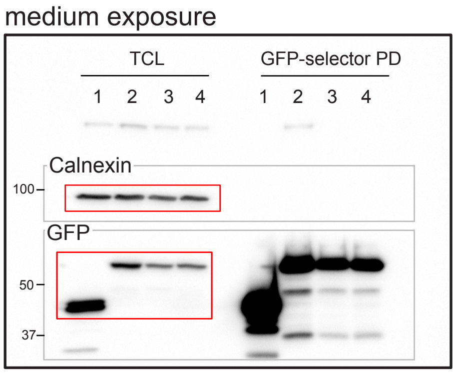

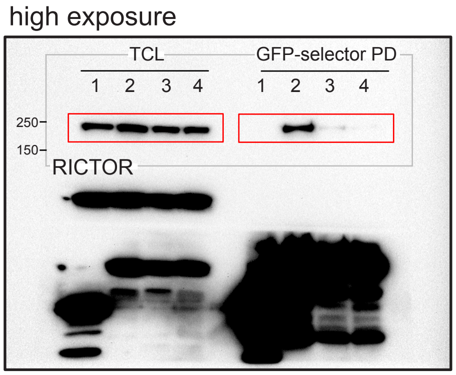

Journal of Cell Science • Supplementary information

Figure S6

A)

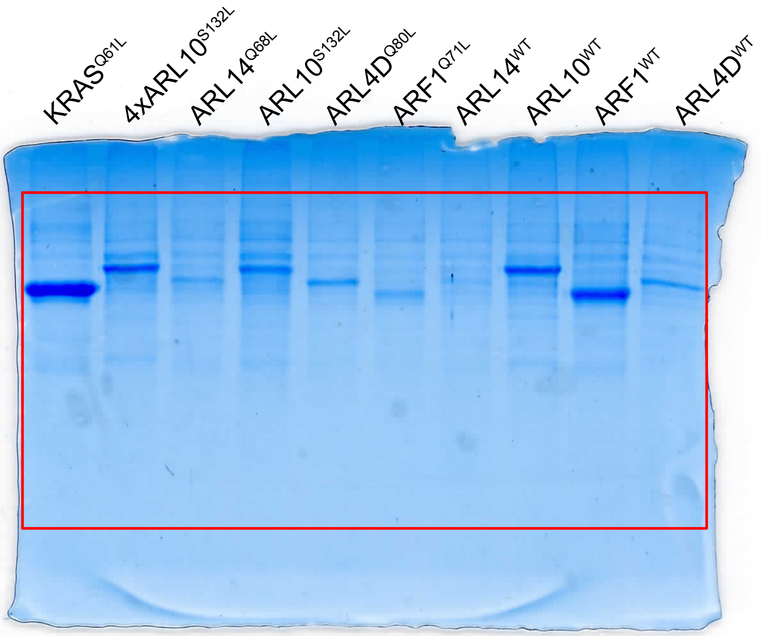

Journal of Cell Science • Supplementary information

Figure S7

F)

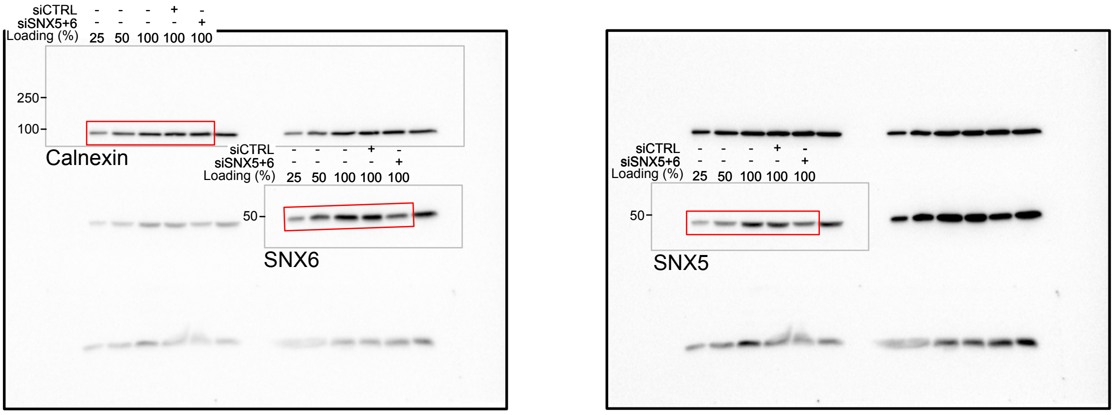

G)

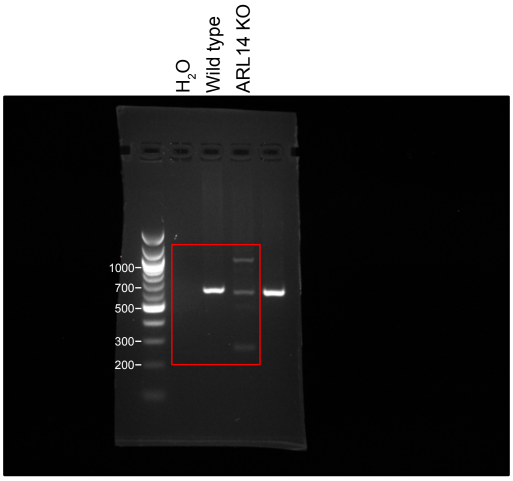

Fig. S8. Blot Transparency

Journal of Cell Science • Supplementary information
